# Proteins that bind regulatory regions identified by histone modification chromatin immunoprecipitations and mass spectrometry

**DOI:** 10.1038/ncomms8155

**Published:** 2015-05-20

**Authors:** Erik Engelen, Johannes H. Brandsma, Maaike J. Moen, Luca Signorile, Dick H. W. Dekkers, Jeroen Demmers, Christel E. M. Kockx, Zehila Ozgür, Wilfred F. J. van IJcken, Debbie L. C. van den Berg, Raymond A. Poot

**Affiliations:** 1 Department of Cell Biology, Erasmus MC, Wytemaweg 80, 3015 CN Rotterdam, The Netherlands; 2 Proteomics Center, Erasmus MC, Wytemaweg 80, 3015 CN, Rotterdam, The Netherlands; 3 Center for Biomics, Erasmus MC, Wytemaweg 80, 3015 CN, Rotterdam, The Netherlands; 4 Francis Crick Institute, Mill Hill Laboratory, The Ridgeway, London NW7 1AA, UK

## Abstract

The locations of transcriptional enhancers and promoters were recently mapped in many mammalian cell types. Proteins that bind those regulatory regions can determine cell identity but have not been systematically identified. Here we purify native enhancers, promoters or heterochromatin from embryonic stem cells by chromatin immunoprecipitations (ChIP) for characteristic histone modifications and identify associated proteins using mass spectrometry (MS). 239 factors are identified and predicted to bind enhancers or promoters with different levels of activity, or heterochromatin. Published genome-wide data indicate a high accuracy of location prediction by ChIP-MS. A quarter of the identified factors are important for pluripotency and includes Oct4, Esrrb, Klf5, Mycn and Dppa2, factors that drive reprogramming to pluripotent stem cells. We determined the genome-wide binding sites of Dppa2 and find that Dppa2 operates outside the classical pluripotency network. Our ChIP-MS method provides a detailed read-out of the transcriptional landscape representative of the investigated cell type.

## Abstract (web summary)

The protein factors that bind to regulatory regions in the genome have not been systematically mapped. Here the authors performed chromatin immunoprecipitations for histone modifications associated with promoters, enhancers or heterochromatin in mouse embryonic stem cells and assigned a genome location to many factors important for pluripotency.

## Graphical abstract

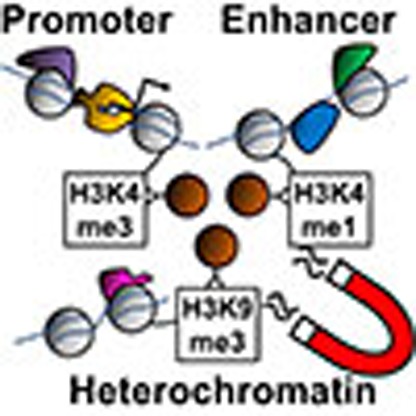

A mammalian genome supports the generation of the hundreds of different cell types in an organism. These cell types display distinct gene expression profiles as a direct consequence of differences in the activation state of their gene promoters and distal *cis*-regulatory elements called transcriptional enhancers. The ENCODE project has generated a wealth of data on the genome-wide chromatin landscape of many different mouse and human cell types^1^,^2^. In particular, the genome-wide identification of regulatory regions such as transcriptional enhancers and promoters and their state of activity has the potential to increase our understanding of how cell type identity is acquired and maintained. From reprogramming experiments it has become increasingly clear that the identity of cells is to a large extent determined by transcription factors, which bind enhancers and promoters^3^,^4^,^5^,^6^. It is therefore of interest to purify native transcriptional enhancers and promoters of a given cell type and identify the proteins that bind to these regulatory regions. Here we performed chromatin immunoprecipitations (ChIP) for histone modifications associated with promoters, enhancers or heterochromatin in mouse embryonic stem cells (ESCs) and identified the proteins present in the different precipitated fractions by mass spectrometry (MS), a method that we named ChIP-MS.

Our ChIP-MS experiments identified 239 factors that we could predict to bind to promoters, enhancers or heterochromatin. Among these factors are subunits of several chromatin-modifying complexes and proteins that play a role in different aspects of transcriptional regulation. We also find key ESC transcription factors such as Oct4, Esrrb, Dppa2 and Klf5 that are not only important for maintaining ESC self-renewal but also facilitate the reprogramming of somatic cells to induced pluripotent stem cells (iPSCs)^4^,^7^,^8^,^9^. Genome-wide data sets were available for 28 ChIP-MS-identified factors and correlated well with the ChIP-MS-based predictions for these factors, suggesting a high level of accuracy of location prediction by ChIP-MS.

For many of the detected factors, the genome-wide localization has not yet been determined and our ChIP-MS results provide the first evidence of their binding preference for a particular type of regulatory DNA. To illustrate that ChIP-MS can identify factors with an interesting genome-wide location, we determined the genome-wide binding sites of pluripotency marker and reprogramming factor Dppa2. We show that Dppa2 is not part of the classical pluripotency transcriptional network and that Dppa2 target genes reach full activation only later in development.

## Results

### ChIP-MS rationale and procedure

Transcriptional enhancers and promoters can be recognized by the chemical modifications of their associated histones, especially histone H3. Promoters of transcribed genes were found to contain histone H3 tri-methylated at lysine 4 (H3K4me3)^10^,^11^ and the level of their activity correlates with the level of H3K27 acetylation (H3K27ac) present^12^,^13^. Enhancers contain histone H3 mono-methylated at lysine 4 (H3K4me1)^14^ and active enhancers can be recognized by the presence of the H3K27ac mark^15^,^16^. Inactive (hetero)chromatin is marked by H3K9me3 (ref. 17). The presence or absence of these and other chromatin marks was used to postulate fifteen different chromatin regions in the mammalian genome, including promoters and enhancers with different levels of activity^13^.

**Figure 1:**
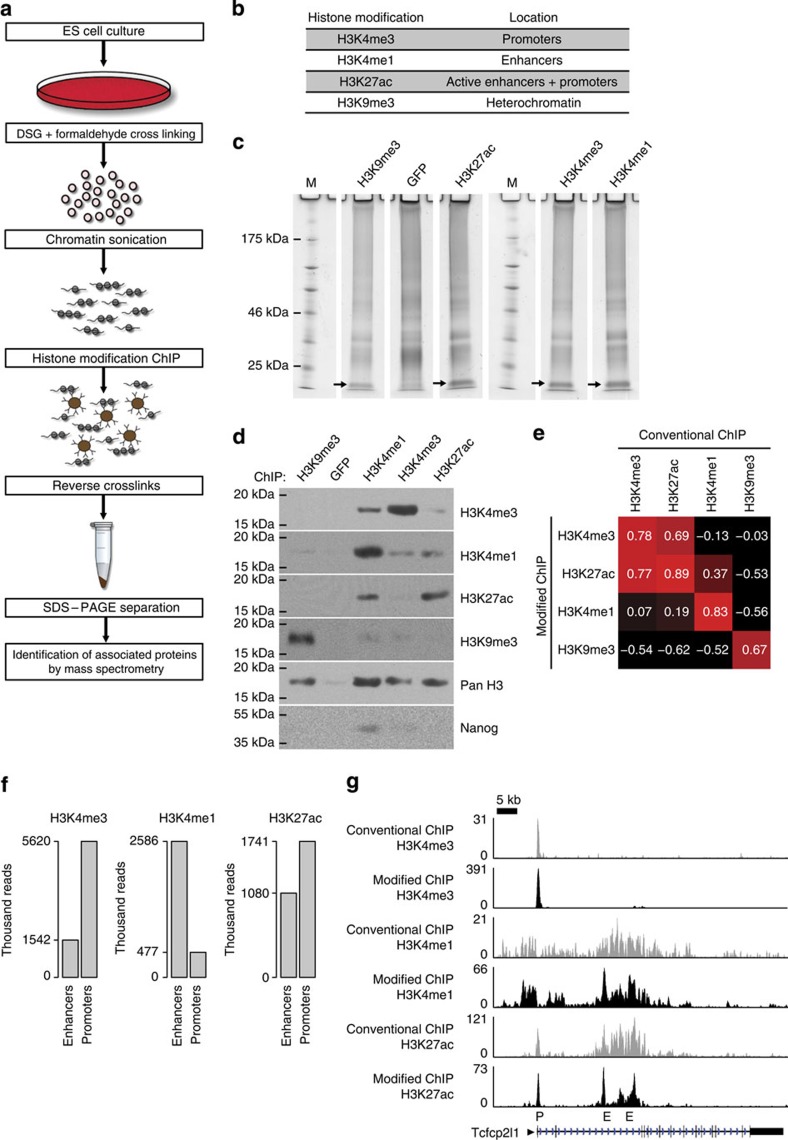
Outline and initial validation of the ChIP-MS protocol. (**a**) Flowchart of the ChIP-MS protocol. (**b**) Histone modifications used in ChIP-MS and their predominant location on the genome. (**c**) Representative 10% polyacrylamide gel with proteins from ChIPs for the indicated histone modifications and the GFP control ChIP. Arrows indicate unresolved histones in the histone modification ChIPs, which are absent in the GFP control ChIP. Molecular weight markers are depicted by M. (**d**) Western blot analyses of the histone modification content, histone content and the presence of Nanog in the immunoprecipitated chromatin fractions. Different ChIPs are indicated at the top, antibodies used for the different western blot analyses are on the right. (**e**) Correlation between DNA regions precipitated by modified ChIP and conventional ChIP for H3K4me3, H3K27ac, H3K4me1 or H3K9me3. (**f**) Overlap of DNA precipitated with modified ChIP for H3K4me3, H3K4me1 or H3K27ac with promoters and enhancers. Number of ChIP-seq reads overlapping with promoters or enhancers is indicated. (**g**) ChIP-seq tracks for modified ChIP or conventional ChIP for H3K4me3, H3K4me1 or H3K27ac around pluripotency gene *Tcfcp2l1*. Sequence reads were plotted relative to chromosomal position. Genome location of *Tcfcp2l1* is shown, scale bar indicates 5 kb of genome. P indicates promoter, E indicates putative enhancer.

We anticipated that ChIP for H3K4me3 would precipitate active promoters and ChIP for H3K4me1 would precipitate enhancers. ChIP for H3K27ac would preferentially precipitate the most active promoters and enhancers, whereas ChIP for H3K9me3 would precipitate heterochromatin. Accordingly, we performed large-scale ChIPs in biological duplicate for H3K4me3, H3K4me1, H3K27ac or H3K9me3, and for green fluorescent protein (GFP) as a control, in mouse ESCs (Fig. 1a,b). Crosslinking of the chromatin was performed with Disuccinimidyl glutarate (DSG), a protein–protein crosslinker, followed by standard formaldehyde crosslinking, to increase the crosslinking efficiency of genome-bound factors to the chromatin^18^,^19^,^20^. ChIP wash steps were performed in low-adherence tubes to increase protein yield and reduce background^20^,^21^. Bound protein factors were de-crosslinked and eluted by prolonged heating in protein denaturing conditions, separated on an SDS–polyacrylamide gel, tryptic peptides isolated and analysed by MS. A representative protein gel showed the (unresolved) histones precipitated with each histone modification antibody but not with the GFP control (Fig. 1c). Analysis by western blot revealed that comparable amounts of chromatin were precipitated in the different histone-modification ChIPs, as indicated by the total content of histone H3 (Fig. 1d). ChIP against H3K4me1, H3K4me3 or H3K27ac precipitated chromatin with these respective histone modifications (Fig. 1d). Minor amounts of H3K4me1 were observed in the H3K4me3 ChIP and vice-versa. This is to be expected as H3K4me1 is present at low levels around active promoters^15^. H3K9me3 ChIP precipitated H3K9me3-marked chromatin but no significant amounts of the other histone modifications (Fig. 1d). We conclude that the histone modification ChIPs efficiently precipitated the intended chromatin fractions.

Subsequently, we tested whether our modified ChIP protocol, which we use for ChIP-MS, still precipitated the intended genomic regions, as compared with conventional ChIP. DNA precipitated by modified ChIP for the different histone modifications was sequenced (ChIP-seq) and mapped to the genome. DNA precipitated by modified ChIP correlated well with the corresponding published conventional ChIP-seq for all four histone modifications (Fig. 1e). Modified H3K4me3 ChIP predominantly precipitated promoters and modified H3K4me1 ChIP precipitated predominantly enhancers, as intended (Fig. 1f). Examples of histone modification tracks around pluripotency genes *Tcfcp2l1* (Fig. 1g) and *Nanog* (Supplementary Fig. 1) show high similarity between our modified ChIP and conventional ChIP. We conclude that the inclusion of additional crosslinker DSG has not significantly altered the genomic regions precipitated by our ChIP protocol, as compared with conventional ChIP.

### Prediction of genome localization of identified factors

We analysed the different precipitated chromatin fractions and GFP control fractions by MS for an unbiased identification of the protein factors present in each fraction. We identified 249 factors that have at least a threefold difference in Exponentially Modified Protein Abundance Index (emPAI) score, a measure for the amount of protein present^22^, in the ChIPs for one histone modification compared with the ChIPs for one or more of the other histone modifications. Included factors should have no or very low presence (more than fivefold lower emPAI score) in any of the GFP control ChIPs (Supplementary Tables 1 and 2, and Supplementary Methods). These two selection steps were included to exclude proteins that bind to chromatin indiscriminately of the tested histone modifications, or are background of the ChIP-MS procedure, respectively. Of the 249 factors, 10 factors were only present in the H3K27ac fraction, which does not discriminate between promoters and enhancers, leaving 239 factors for which we could predict their binding to promoters, enhancers or heterochromatin. We assigned to identified factors the locations ‘promoter', ‘enhancer' and ‘heterochromatin' according to the fraction (H3K4me3, H3K4me1 and H3K9me3, respectively) in which they have the highest emPAI value (Supplementary Tables 1, 2 and 3, and Supplementary Methods). This annotation is not absolute, as factors can be present in more than one location, but it does provide clarity and facilitates a more systematic validation with published genome-wide localization data (see below).

We also indicated the presence of a factor in the H3K27Ac fractions by calculating the ratio of its average emPAI value in the H3K27ac fractions over its H3K4me3 emPAI score or its H3K4me1 emPAI score, whichever one is the highest, a ratio that we call the H3K27ac ratio. Presenting the ChIP-MS association of a factor with the H3K27ac modification in this way compensates for the considerable differences in ChIP-MS detection levels for different proteins (Supplementary Table 1) and is therefore more informative than its H3K27ac emPAI value *per se*. Small emPAI values for H3K4me3 or H3K4me1 increase the uncertainty of the H3K27ac ratio value. We used the H3K27ac ratio as a predictor for the level of H3K27ac, and thereby the activity, of the promoters or enhancers bound by the factor (Supplementary Table 1).

**Figure 2:**
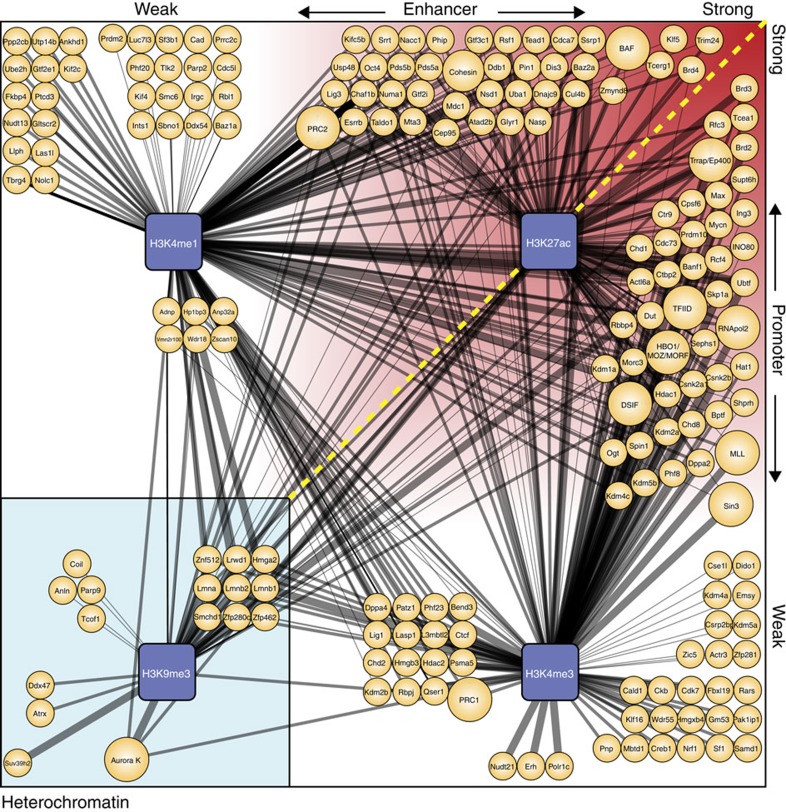
ChIP-MS predicted locations of identified factors and complexes. Visual representation of factors (small orange circles) and complexes (large orange circles) identified by ChIP-MS for four different histone modifications (blue squares). Thickness of the edges indicates average emPAI score of a factor or complex in histone modification ChIP. Factors and complexes are positioned according to their ChIP-MS location prediction. To the left of the yellow dashed line are predicted enhancer binders, positioned horizontally from weak activity enhancers (left) to strong activity enhancers (right) according to their H3K27ac ratio. To the right of the yellow dashed line are predicted promoter binders positioned vertically from weak activity promoters (bottom) to strong activity promoters (top) according to their H3K27ac ratio. In the left bottom square are factors and complexes predicted to bind heterochromatin.

For a visual representation (Fig. 2), identified factors and complexes were allocated according to their predicted binding to promoters, enhancers or heterochromatin. The calculated H3K27ac ratios were used to position predicted enhancer-binding factors on the upper horizontal axis reflecting the level of activity of bound enhancers or position-predicted promoter binders on the right vertical axis reflecting the level of activity of bound promoters (Fig. 2).

An early indication that the predictions from our ChIP-MS experiments were valid came from the ‘promoter' prediction of all five identified RNApol2 subunits and seven identified TFIID subunits (Supplementary Table 3). The H3K9 methyltransferase Suv3-9 binds pericentric heterochromatin^23^ and was indeed observed solely in the H3K9me3 fraction (‘heterochromatin' prediction, Fig. 2 and Supplementary Table 1). We identified a large number of subunits of established chromatin-modifying complexes including the BAF complex, Sin3 complex and MLL complex (Fig. 2 and Supplementary Table 3). Strikingly, the localization prediction for different subunits within the same complex was nearly 100% identical (Supplementary Table 3), indicating a high level of consistency in the predictions.

Among the ChIP-MS identified factors with the highest H3K27ac ratio, predicting binding to highly active regulatory regions, were chromatin factors of the BET family: Brd2, 3 and 4 (Fig. 2 and Supplementary Table 1). BET family members were shown to bind to hyperacetylated chromatin^24^. Brd4 was recently identified as a key factor in the marking and functional maintenance of exceptionally large and active ‘super enhancers', which regulate the expression of cell fate-determining genes^25^,^26^. ChIP-MS classified Brd4 to bind predominantly to enhancers and Brd2 and 3 to bind promoters. Using ChIP-seq, Brd2 and Brd3 were indeed shown to bind promoters, whereas Brd4 was also present at enhancers^27^. The finding that from all ChIP-MS detected proteins, several BET family members are among the proteins with the highest H3K27ac ratio, validates the use of the H3K27ac ratio as an indicator of the level of activity of bound promoters or enhancers.

Many studies have identified factors that are important for maintaining ESC pluripotency^28^,^29^,^30^ or factors that reprogramme somatic cells towards ESC-like iPSCs^4^,^6^,^7^. We found that more than a quarter of our ChIP-MS identified factors (63 out of 239) have a role in pluripotency acquisition or maintenance (Supplementary Table 1). ChIP-MS-identified factors included established reprogramming factors Oct4, Esrrb, Klf5 and Mycn (Fig. 2 and Supplementary Table 1), which as part of a 3–4 factor mix, reprogramme somatic cells to iPSCs^4^,^7^,^8^. Our ChIP-MS data predicted that Oct4, Esrrb and Klf5 bind predominantly to enhancers and Mycn predominantly binds to promoters, in agreement with published genome localization data^3^,^31^. Nanog, another well-known pluripotency factor, is difficult to detect by MS^21^,^32^ and was indeed not identified by ChIP-MS. Western blot analysis of our ChIP-MS samples showed that Nanog was present in the H3K4me1 fraction (Fig. 1d), suggesting it binds to enhancers, in agreement with published data^3^.

### Estimation of ChIP-MS prediction accuracy

**Figure 3:**
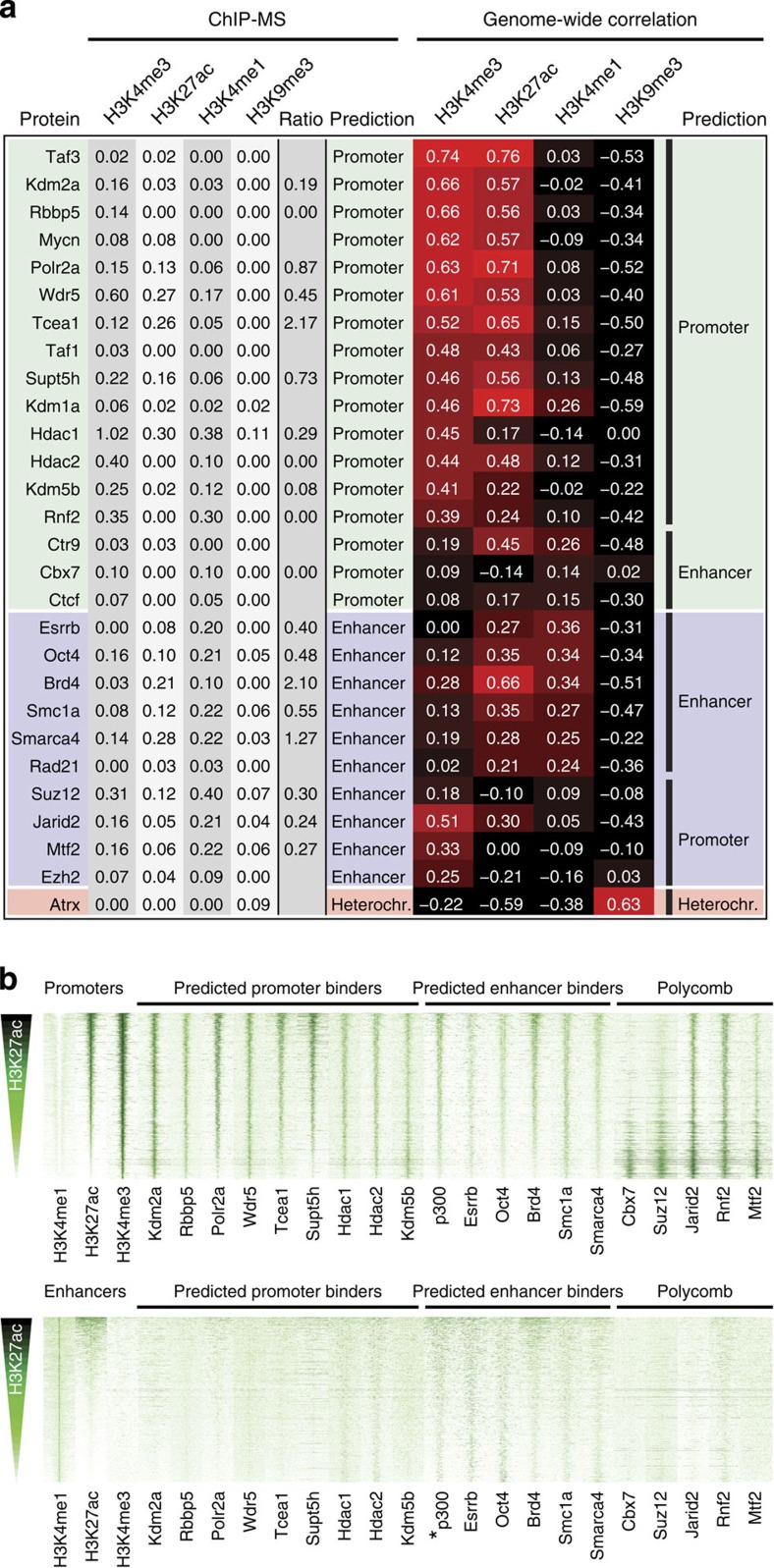
Validation of ChIP-MS predictions with published genome-wide location information. (**a**) Comparison of location prediction by ChIP-MS with location prediction by correlation of genome-wide binding sites with the indicated histone modifications on the genome. Protein factors for which genome-wide locations in mouse ESCs are determined by ChIP-seq are listed on the left, according to their ChIP-MS prediction as promoter binder (top, green panel), enhancer binder (middle, blue panel) or heterochromatin binder (bottom, red panel). Indicated in columns from left to right are: protein factor, its average emPAI values in the different histone modification ChIPs, its H3K27ac ratio (if highest emPAI value⩾0.1), ChIP-MS location prediction, correlation of genome-wide binding sites with the indicated histone modifications and location prediction by highest correlation with a histone modification, according to Fig. 1b. (**b**) Binding of selected protein factors to promoters and enhancers in mouse ESCs. Heatmaps of 12,913 promoters (upper panel) or 30,564 enhancers (lower panel), centred on H3K4me3 signal (Promoters) or H3K4me1 signal (Enhancers), ranked on H3K27ac content from top to bottom. Displayed is 8 kb around the centre of the promoter or enhancer. Normalized ChIP-seq reads representing the level of H3K4me1, H3K27ac and H3K4me3 histone modifications are indicated in the first three lanes. Normalized ChIP-seq reads representing relative binding intensity to promoters (upper panel) and enhancers (lower panel) of protein factors from **a** (highest emPAI value ⩾0.1) are displayed in lanes 4–12 and 14–20. Factors are arranged according to binding prediction or Polycomb factor identity. *p300 was not predicted by ChIP-MS but its genome-wide location was included in lane 13 for comparison.

Our list of 239 ChIP-MS assigned factors includes 28 factors for which the genome-wide binding sites have been determined in mouse ESCs by ChIP-seq (Fig. 3a), which provides an opportunity to probe the accuracy of our localization prediction. In a first analysis, we compared our ChIP-MS-based predictions (Fig. 3a) with location predictions derived from the correlation of factor binding sites with the different histone marks on the genome (Fig. 3a). Of the 17 factors predicted by ChIP-MS to be promoter associated, 14 factors (82%) were indeed most associated on the genome with the active promoter mark H3K4me3 (Fig. 3a). In the case of Ctr9, Ctcf and Cbx7, the ChIP-MS prediction were not conform the location prediction by genome-wide correlation. However, in these cases both the ChIP-MS values and genome-wide correlations for H3K4me3 and H3K4me1 (which differentiates between ‘promoter' and ‘enhancer' prediction) were very similar (Fig. 3a). Moreover, the correlation with any of the four tested histone marks was low for Ctcf and Cbx7.

Of the ten factors predicted by ChIP-MS to be predominantly associated with enhancers, six had indeed the highest association on the genome with enhancer mark H3K4me1 (Fig. 3a). These include Oct4 and Esrrb, two key pluripotency and reprogramming transcription factors, and Smarca4 (Brg1), the catalytic subunit of the SWI-SNF chromatin-modifying complex (Fig. 3a). All the wrongly annotated factors (Suz12, Jarid2, Mtf2, Ezh2) are members of the Polycomb family of repressor proteins, which had ‘Enhancer' ChIP-MS predictions but were assigned ‘Promoter' by the genome-wide correlation of their binding sites (Fig. 3a). Polycomb factors are abundant in ESCs and often bind broadly at relatively inactive promoters with similarly low levels of H3K4me3 and H3K4me1 (see below)^33^,^34^. Both histone marks are indeed faithfully detected by ChIP-MS but in the above cases this leads to wrong predictions, albeit by small margins in the H3K4me3 and H3K4me1 ChIP-MS values (Fig. 3a). Atrx was correctly assigned by ChIP-MS to bind H3K9me3-containing heterochromatin (Fig. 3a). We conclude from this analysis that ChIP-MS predicted factor location to promoters, enhancers or heterochromatin with high accuracy, with the few false identifications in the expected grey areas.

Subsequently, we assessed whether the H3K27ac ratio of a factor correlates with the association of its binding sites with H3K27ac on the genome (Fig. 3a). From the factors with genome-wide location information (Fig. 3a), we took factors with a highest emPAI value of 0.1 or higher, to be well above the detection limit of our ChIP-MS experiments. We calculated for each of these factors their H3K27ac ratio (Fig. 3a). These ratios were compared with the correlation of genome-wide binding of these factors with H3K27ac-marked regions (Fig. 3a). Indeed, promoter-predicted factors Tcea1, Polr2a and Supt5h have the highest H3K27ac ratios and have relatively high correlations with H3K27ac on the genome (Fig. 3a). Promoter-predicted factors Hdac1 and 2, Kdm5b, Rnf2 and Cbx7 have low H3K27ac ratios and are factors with relatively low genome-wide associations with H3K27ac (Fig. 3a). Kdm2a and Rbbp5 have low H3K27ac ratios but still have high genome-associations with H3K27ac. Hence, in these two cases, the H3K27ac ratios do not correlate well with H3K27ac association on the genome. Brd4 is the enhancer-predicted factor with the highest H3K27ac ratio and indeed has the highest correlation with H3K27ac on the genome (Fig. 3a). Oct4, Esrrb and Smc1a have intermediate H3K27ac ratios and intermediate genome-wide association with H3K27ac. Smarca4 has a high H3K27ac ratio, which in this case was not a good predictor, as Smarca4 has an intermediate level of genome-wide association with H3K27ac. We conclude from our above analyses that H3K27ac ratios provide a good, albeit not flawless, indication of the level of localization to H3K27ac-marked regions on the genome.

In a second approach to assess the accuracy of ChIP-MS, we investigated the presence of the above factors at promoters and enhancers in mouse ESCs. We assigned promoters as being present at the start of a gene and containing H3K4me3. Enhancers were assigned by their H3K4me1 content in the absence of H3K4me3. Promoters and enhancers were ranked top to bottom by their H3K27ac content (Fig. 3b). Promoter-predicted factors Kdm2a, Rbbp5, Polr2a, Wdr5, Tcea1, Supt5h and Kdm5b were indeed observed to only bind promoters and not enhancers (Fig. 3b). Hdac1 and Hdac2 predominantly bound to promoters but also showed binding to enhancers. Enhancer-predicted factors Esrrb, Oct4, Brd4 and Smc1a all showed strong binding to enhancers (Fig. 3b). These factors also showed binding to promoters to varying degrees. For comparison, we also included published genome-wide localization data of archetypal enhancer binder p300 in Fig. 3b. Remarkably, p300 showed binding to enhancers and promoters (Fig. 3b), as previously observed in human ESCs^33^. Polycomb factors Rnf2, Jarid2, Suz12, Cbx7 and Mtf2 bind promoters with no correlation (Rnf2, Jarid2) or an anti-correlation (Cbx7, Suz12 and Mtf2) for H3K4me3 and H3K27ac content (Fig. 3b). The broad binding of Polycomb factors to promoters with relatively low H3K4me3 levels would explain their similar ChIP-MS values for H3K4me3 and H3K4me1 (Fig. 3a) and the associated ChIP-MS prediction uncertainties. The above analysis suggests that ChIP-MS-mediated prediction of binding to promoters or enhancers has high accuracy, with Polycomb factors again being an exception.

### Dppa2 is not part of the classical ESC pluripotency network

**Figure 4:**
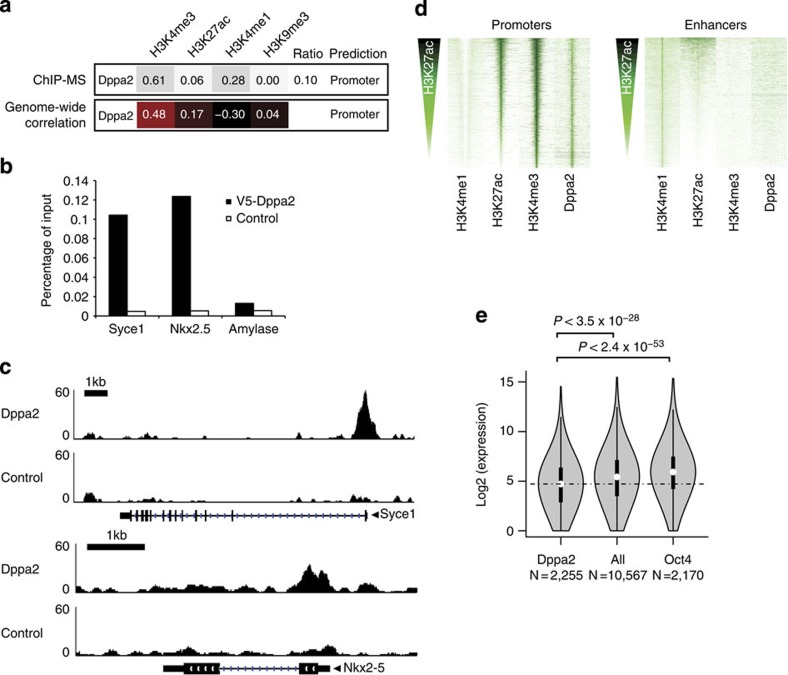
Analyses of genome-wide binding sites of Dppa2. (**a**) Comparison of location prediction for Dppa2 by ChIP-MS with location prediction by the correlation of identified Dppa2 genome-wide binding sites with the indicated histone modifications on the genome. Indicated in the upper panel from left to right are: Dppa2 average emPAI values in the different histone modification ChIPs, its H3K27ac ratio and ChIP-MS location prediction. Indicated in the lower panel from left to right are: the correlation of Dppa2 genome-wide binding sites with the indicated histone modifications and location prediction by highest correlation with a histone modification, according to Fig. 1b. (**b**) Binding of Dppa2 to the promoters of the indicated genes, detected by anti-V5 ChIP on V5-Dppa2-expressing ESCs or control ESCs. Precipitated DNA for the indicated genes is shown as percentage of input, the *Amylase* gene is used as a negative control region. (**c**) Localization of Dppa2 on the promoter of *Syce1* (upper panel) or *Nkx2*–*5* (lower panel). Sequence reads from anti-V5 ChIP-seq on V5-Dppa2-expressing ESCs (Dppa2) or control ESCs (Control) were plotted relative to chromosomal position. Genome locations of *Syce1* gene (upper panel) and *Nkx2*–*5* gene (lower panel) are shown, scale bars indicate 1 kb of genome. (**d**) Binding of Dppa2 to promoters and enhancers in mouse ESCs. Heatmaps of 12,913 promoters (left panel) or 30,564 enhancers (right panel), centred on H3K4me3 signal (Promoters) or H3K4me1 signal (Enhancers), ranked on H3K27ac content from top to bottom. Displayed is 8 kb around the centre of the promoter or enhancer. Normalized ChIP-seq reads representing the level of H3K4me1, H3K27ac and H3K4me3 histone modifications are indicated in the first three lanes. Normalized V5-Dppa2 ChIP-seq reads representing the relative binding intensity of Dppa2 to promoters (left panel) and enhancers (right panel) are displayed in the fourth lane of each panel. (**e**) Distribution of absolute expression levels of H3K4me3 marked genes in mouse ESCs that (from left to right) are bound at the promoter by Dppa2, all genes, and bound within 20 kb around the promoter by Oct4. Shown is a violin plot where the white dot indicates the median and the thick black bar indicates 50% of the genes. Log2 value of the absolute expression, derived from published RNAseq data, the number of genes in each category and *P*-values by Mann–Whitney test are indicated.

For many of the ChIP-MS detected factors, the genome localization has not yet been determined by genome-wide ChIP and our experiments provide the first information on their genome binding preferences. As an example that ChIP-MS can identify factors with an unusual and therefore interesting genomic distribution, we focused on Dppa2 (Developmental PluriPotency Associated 2). Dppa2 is a member of family that also contains Dppa3 (Stella) and Dppa4, which all harbour a SAP DNA-binding domain. The genome-wide binding sites of members of this family have not been determined so far. Dppa2 is exclusively expressed in the inner cell mass of the early embryo and later in the developing germ line and in cells derived from these tissues, such as ESCs and primordial germ cells^35^. Furthermore, Dppa2 expression was identified as an early marker for successful reprogramming towards iPSCs^9^. Dppa2 knockout ESCs have a slower proliferation rate and Dppa2 knockout mice die after birth from respiratory defects^36^. Recently, it was shown that Dppa2, in combination with Lin28, Sall4 and Esrrb, drives the reprogramming of fibroblasts into iPSCs^9^. We selected Dppa2 because its ChIP-MS profile was unusual for a pluripotency inducing factor. Dppa2 had the highest emPAI score for H3K4me3 but a low H3K27ac ratio, suggesting it binds predominantly to promoters with low activity (Fig. 4a). This is different for other pluripotency-inducing factors, such as Oct4 and Esrrb, which predominantly bind moderately active enhancers (Fig. 3b). To identify the genome-wide binding sites of Dppa2, we established an ESC line that expressed V5-tagged Dppa2 and we performed anti-V5 Dppa2 ChIP and sequenced the precipitated genomic DNA (ChIP-seq). We verified that V5-Dppa2 binds to the promoters of *Syce1* and *Nkx2*–*5* (Fig. 4b,c), the only known Dppa2 genomic binding sites^36^, which suggested that our V5-Dppa2 ChIP identified bona-fide Dppa2-binding sites. Dppa2-binding sites had the highest genome-wide association with H3K4me3, whereas the association with H3K27ac (Fig. 4a) is lower than that of Oct4 and Esrrb (Fig. 3a). Subsequently, we investigated the presence of Dppa2 at promoters and enhancers in mouse ESCs. We found that Dppa2 binds promoters but is absent from enhancer regions (Fig. 4d). Interestingly, Dppa2 promoter binding displayed no correlation with H3K27ac content (Fig. 4d), a binding pattern that otherwise was only observed with the repressor Kdm5b and the Polycomb repressor proteins (Fig. 3b). These analyses suggest that our Dppa2 ChIP-MS location prediction was correct and that the binding pattern of Dppa2 is different compared with other reprogramming factors.

**Figure 5:**
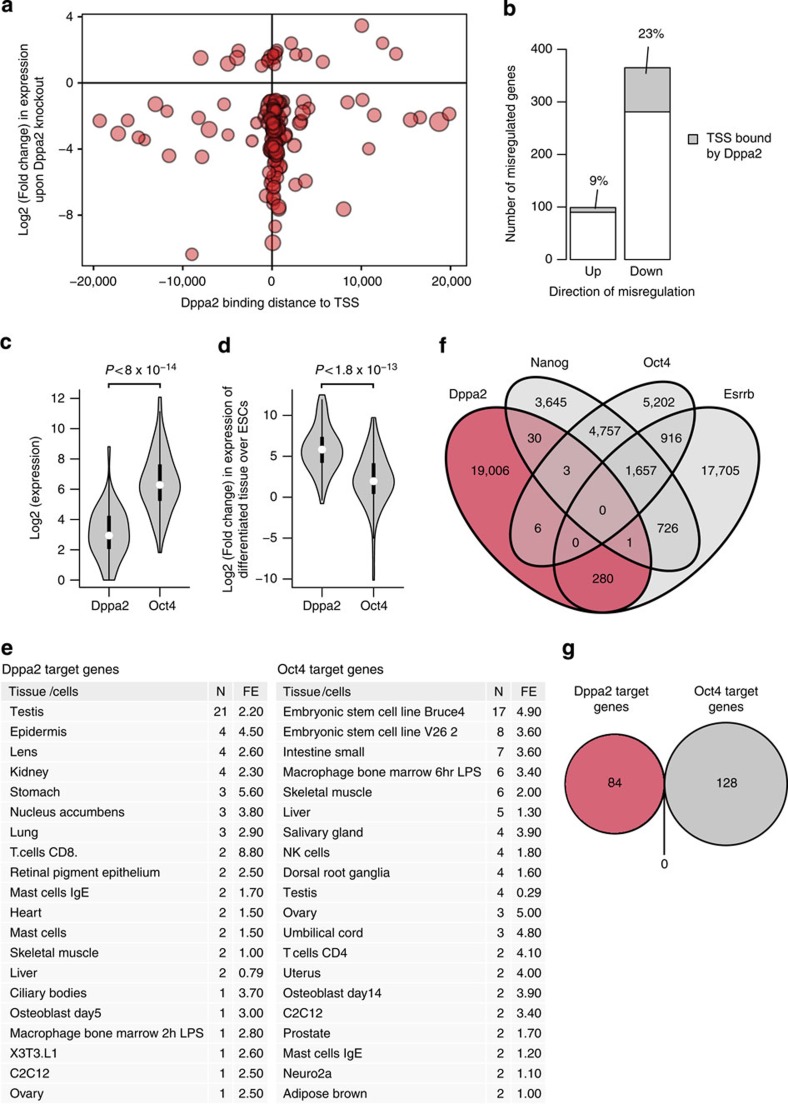
Dppa2 target genes and their overlap with the pluripotency network. (**a**) Bubble plot indicating the positions of Dppa2-binding sites relative to the transcription start site (TSS) on genes that are either upregulated ⩾2-fold (upper part) or downregulated ⩾2-fold (lower part) upon Dppa2 gene knockout in mouse ESCs. Log2 of the fold change in expression upon Dppa2 KO is indicated on the *y*-axis. Distance of Dppa2-binding sites from the TSS is indicated on the *x*-axis. Size of the bubbles correlates with fold difference of Dppa2 ChIP peak over control. (**b**) Bar diagram showing the total number of upregulated and downregulated genes upon Dppa2 knockout and the number of these genes bound by Dppa2 within 1 kb from the TSS (grey areas). The number of Dppa2 bound genes is also indicated as a percentage of the total number of upregulated or downregulated genes. (**c**) Distribution of absolute expression levels in mouse ESCs of Dppa2 target genes and Oct4 target genes. Dppa2 target genes are bound by Dppa2 within 1 kb of the TSS and ⩾2-fold downregulated upon Dppa2 knockout, Oct4 target genes are bound by Oct4 within 20 kb of the TSS and ⩾2-fold downregulated after 24 h of Oct4 depletion. Shown is a violin plot where the white dot indicates the median and the thick black bar indicates 50% of the genes. Log2 of the absolute expression, derived from published RNAseq data and *P*-value by Mann–Whitney test are indicated. (**d**) Distribution of the fold change in expression of Dppa2 target genes and Oct4 target genes in the differentiated tissue with the highest expression versus expression in mouse ESCs. Shown is a violin plot where the white dot indicates the median and the thick black bar indicates 50% of the genes. Log2 of the fold change in expression, derived from published RNAseq data and *P*-value by Mann–Whitney test are indicated. (**e**) Lists of tissues or cells where Dppa2 target genes (left panel) or Oct4 target genes (right panel) are highest expressed. Tissue or cells, the number of genes (N) and fold enrichment (FE) of a tissue/cell type within Dppa2 target genes or Oct4 target genes are indicated. The 20 tissues/cells with the highest number of gene overlap and fold enrichment are shown. (**f**) Venn diagram showing the overlap of genomic binding sites in mouse ESCs of Dppa2, Nanog, Oct4 and Esrrb. (**g**) Venn diagram showing the lack of overlap of Dppa2 target genes and Oct4 target genes.

As expected, Dppa2 binds the promoters of genes with a lower median expression than other H3K4me3-marked promoters and Oct4-bound genes in ESCs (Fig. 4e and Supplementary Dataset). The gene expression profile of Dppa2 knockout ESCs was recently determined and it was observed that far more genes were downregulated than upregulated, compared with wild-type ESCs^36^. Dppa2 binds the promoters of nearly a quarter of the downregulated genes but much less to promoters of the upregulated genes (Fig. 5a,b). This suggests that Dppa2 maintains the expression of its putative target genes (Supplementary Table 4) by binding at their promoter. In contrast, Oct4 maintains the expression of its target genes by binding mostly outside promoters (Supplementary Fig. 2). We find that the median expression of Dppa2 target genes is tenfold lower than the median expression of Oct4 target genes in ESCs (Fig. 5c). Nearly, all Dppa2 target genes are higher expressed in tissues other than ESCs (Fig. 5d and Supplementary Table 4). The largest minority of Dppa2 target genes is highest expressed in testes, but many Dppa2 target genes have their highest expression in other tissues (Fig. 5e and Supplementary Table 4). For Oct4, the pattern is very different, as many Oct4 target genes have their highest expression in ESCs (Fig. 5d,e). Considering that Dppa2 and Oct4 appear to regulate different sets of genes, we determined the overlap in genome-wide binding sites between classical pluripotency factors such as Oct4, Nanog and Esrrb, and Dppa2. Whereas Oct4, Nanog and Esrrb showed an extensive overlap in binding sites, as previously reported^3^, Dppa2 showed little overlap with these factors (Fig. 5f). In particular, the overlap of Dppa2 with Oct4 and Nanog was nearly absent. Moreover, there was no overlap between Dppa2 target genes and Oct4 target genes (Fig. 5g). We conclude that Dppa2 regulates a set of genes that is separate from the set of genes regulated by the classical pluripotency transcription factors, and not ESC specific in its expression.

## Discussion

We describe here ChIP-MS, a method to predict the binding of factors to enhancers or promoters. Our experimental setup is straightforward and does not rely on metabolic labelling for quantification by MS. Nevertheless, using emPAI^22^ and the simple rule that the ChIP fraction in which a factor has the highest emPAI score decides its binding prediction, we achieved a remarkably high percentage of correct predictions when comparing to published ChIP-seq data. We employed crosslinking with protein–protein crosslinker DSG, followed by standard crosslinking with formaldehyde. We previously used this ‘double crosslinking' procedure to improve ChIP efficiency^19^,^20^,^21^. Aside from our extended crosslinking procedure, our ChIP-MS procedure is based on standard ChIP protocols and MS procedures, which should facilitate its application in other cell types.

A number of studies have been performed to screen for protein factors that bind to individual histone modifications by using modified histone peptides^37^,^38^,^39^ or *in vitro* assembled modified nucleosomes^40^, or identify protein factors that bind native chromatin harbouring specific histone modifications by conventional ChIP combined with mass spectroscopy^41^. Binding to different tri-methylated lysines was assessed in these studies, but binding to enhancer marks or activity marks, such as the H3K4me1- or H3K27ac-marked chromatin that we interrogated with ChIP-MS, has not been addressed yet. Previous studies identified a number of ubiquitously expressed factors that we also observed in our ChIPs. However, our ChIP-MS procedure is sufficiently sensitive to also detect the sequence-specific transcription factors, such as Oct4, Esrrb and Klf5, that determine ESC identity. These factors will be different in other cell types, which makes our procedure highly suitable to study the changing spectrum of regulatory region-associated factors during cell differentiation.

The list of proteins for which we predict genome localization by ChIP-MS (Fig. 2 and Supplementary Table 1) contains factors that play a role in a number of cellular processes, including all levels of transcriptional regulation and chromatin organization. ChIP-MS detected chromatin-modifying complexes with clear location predictions, such as the BAF complex (enhancer), Aurora kinase complex (heterochromatin), Trrap complex (promoter), MLL complex (promoter) and Sin3 complex (promoter). The annotation of subunits of the Polycomb complexes PRC1 and PRC2 was more ambiguous, as PRC1 and PRC2 bind broad areas around inactive promoters marked to a similar extent by H3K4me3 and H3K4me1, leading to false ‘enhancer' predictions for several PRC subunits. Fortunately, Polycomb factors are well characterized^34^,^42^ and would therefore be easy to recognize and treated with caution in any ChIP-MS prediction list.

Figure 2 ranks detected factors by the activity of their bound promoters or enhancers. To our knowledge, such an analysis has not been performed yet and provides a read-out on an important criterion for a large set of factors in ESCs. The ranking is based on the H3K27ac ratio; the ratio of the H3K27ac emPAI score over the highest of the H3K4me3 emPAI score or H3K4me1 emPAI score, which we anticipated would be the more informative value than the H3K27 emPAI score *per se*, as it compensates for the considerable differences in ChIP-MS detection levels for different factors. Perhaps the clearest indication that the H3K27ac ratio performs well in differentiating factors by the activity of their bound regions is that out of the nearly 240 factors detected by ChIP-MS, 3 members of the family of BET proteins, including Brd4, are among the proteins with the highest H3K27ac ratios. Brd4 was recently identified as a functional component and marker of ‘super enhancers'^25^,^26^, arguably the most active enhancers in the genome of a cell. The ranking of the chromatin-modifying complexes follows common sense. The activating BAF chromatin remodelling complex and Trrap histone acetylase complex have higher H3K27ac ratios than the Sin3 repression complex and the PRC1 and PRC2 complexes. The good performance of ChIP-MS on factors with a known genome-wide location suggests that also the localization predicted for the many factors without genome-wide ChIP data will in most cases be accurate. Our data set, graphically represented in Fig. 2, therefore provides valuable new information on the transcriptional network in ESCs. Importantly, ChIP-MS can detect factors with an unusual, and therefore interesting, genome localization that can then be further investigated, Dppa2 being an example.

We performed our ChIP-MS experiments in ESCs. The establishment and maintenance of pluripotency, as well as the exit from pluripotency, is intensely studied in ESCs and several large data sets of relevant factors for the above processes are available. We find that a quarter (63 factors) of the ChIP-MS-detected factors contributes to maintaining pluripotency. ChIP-MS detected Oct4, Esrrb, Klf5, Dppa2 and Mycn, factors which, as part of a 3–4 factor mix, reprogramme somatic cells to iPSCs^4^,^7^,^8^. Intriguingly, ChIP-MS predicts that these factors do not all bind to the same type of locations on the genome. Oct4 and Esrrb were predicted to bind moderately active enhancers, whereas Klf5 binds to highly active enhancers. Mycn and Dppa2 were predicted to bind to high and low activity promoters, respectively. This suggests a division of labour between the different factors in the reprogramming process.

We found that Dppa2 had a different ChIP-MS profile compared with other pluripotency factors and accordingly we determined its genome-wide binding sites by ChIP-seq. Indeed, Dppa2 turned out to be an unusual pluripotency factor. Dppa2-binding sites and target genes do not overlap with Oct4, suggesting that Dppa2 is not part of the classical pluripotency circuit. Furthermore, Dppa2 target genes were found to be much lower expressed than Oct4 target genes in ESCs and higher expressed later in development. It is an intriguing question how Dppa2 can be an early marker and factor for reprogramming to iPSCs, as was recently shown^9^, without actually regulating ESC-specific genes. Dppa2 was proposed as a factor that binds target genes to maintain an active chromatin structure and facilitate their later expression^36^. This epigenetic marking hypothesis is consistent with the expression pattern of the identified Dppa2 target genes. However, we did not find that genes bound by Dppa2 in ESCs were preferentially downregulated in Dppa2 knockout lungs, using a published gene expression set^36^.

In conclusion, we established here a method to annotate factors to enhancers and promoters with different activities. ChIP-MS is straightforward in its setup, which should facilitate its application to other cell types and growth conditions, provided sufficient cell quantities can be obtained. We showed that ChIP-MS data add to our knowledge and understanding of the transcriptional circuitry that determines cell identity.

## Methods

### Cell lines and constructs

Mouse embryonic stem cell line CGR8 was grown on gelatin-coated dishes without feeders in Glasgow Minimum Essential Medium (GMEM) supplemented with leukemia inhibitory factor (LIF), 15% fetal bovine serum, 0.25% sodium bicarbonate, 1 mM glutamine, 1 mM sodium pyruvate, non-essential amino acids, 50μM β-mercaptoethanol and penicillin/streptomycin, as previously described^19^. The coding sequence for Dppa2 was amplified from mouse ES cell cDNA and cloned with an N-terminal V5-tag into a pPyCAG-driven expression vector. CGR8 cells were transfected with the V5-Dppa2 expression vector using Lipofectamine 2,000 (Invitrogen), clones were selected with 1 μg ml^−1^ puromycin (Sigma) and stable expression of V5-tagged Dppa2 tested by western blot analysis with anti-V5 antibody (1:2,000; Invitrogen).

### ChIP-MS procedure

For each histone modification ChIP, 300 × 10^6^ ESCs were used. For chromatin preparation, cells were washed on plate three times with PBS and incubated with 2 mM DSG (Thermo Scientific) in PBS for 45 min at room temperature. Subsequently, ESCs were washed in PBS three times, 0.1 volume of 11% formaldehyde (Merck) in 50 mM HEPES-KOH (pH 7.5), 100 mM NaCl, 1 mM EDTA, 0.5 mM EGTA was added, mixed and incubated for 12 min at room temperature, washed two times in 4 °C PBS and collected by centrifugation. All subsequent steps were performed on ice with pre-cooled buffers. Cell lysis was performed as described^43^. In brief, cells were collected and resuspended in LB1 (50 mM HEPES-KOH, pH 7.5, 140 mM NaCl, 1 mM EDTA, 10% glycerol, 0.5% NP-40, 0.25% Triton X-100). After 10 min of incubation, cells were pelleted by centrifugation and resuspended in LB2 (10 mM Tris-HCl, pH 8.0, 200 mM NaCl, 1 mM EDTA, 0.5 mM EGTA). After 10 min of incubation, cells were pelleted and resuspended in 3 ml of freshly prepared LB3 (10 mM Tris-HCl, pH 8.0, 100 mM NaCl, 1 mM EDTA, 0.5 mM EGTA, 0.1% Na-deoxycholate, 0.5% N-lauroylsarcosine) and sonicated on a Soniprep 150 (MSE), 27 cycles 15 s on, 45 s off on amplitude 7. After sonication, enriched DNA fragment size was confirmed to be between 200 and 1,000 bp. 300 × 10^6^ ESCs yielded approximately 10 mg chromatin (as measured by DNA content).

Antibodies used in the different histone modification ChIPs or GFP control ChIP are against H3K4me1 (ab8895, Abcam), H3K4me3 (ab8580, Abcam), H3K27Ac (ab4729, Abcam), H3K9me3 (ab8898, Abcam) and GFP (sc8334, Santa Cruz Biotechnology). To prevent immunoglobin elution and subsequent interference with the MS analysis, 50 μg antibodies were crosslinked to 500 μl Protein A magnetic bead solution (15 mg beads, Life Technologies) with Dimethyl Pimelimidate (Sigma). Crosslinked antibody–bead complexes were equilibrated in LB3 buffer and subsequently blocked with 0.5 mg ml^−1^ BSA (New England Biolabs) and 0.2 mg ml^−1^ sonicated salmon sperm DNA (Stratagene) for 1 h. The antibody–bead mixture was rotated overnight with ∼10 mg chromatin at 4 °C. Beads were transferred to 1.5 ml no stick tubes (Alpha laboratories) and washed five times for 5 min in RIPA buffer (50 mM HEPES-KOH (pH 7.6), 500 mM LiCl, 1 mM EDTA, 1% NP-40, 0.7% Na-deoxycholate). After washing, the beads were boiled for 35 min at 95 °C in 2 × SDS sample buffer (100 mM Tris-HCl (pH 6.8), 200 mM DTT, 4% SDS, 20% Glycerol, 0.2% Bromophenol blue) and supernatant was transferred to a fresh tube. ChIP-MS samples were run on 10% precast SDS–PAGE gels (NuPage Invitrogen) and stained with colloidal Coomassie stain (Invitrogen). Gel lanes were sliced, in-gel digested with trypsine to yield peptides and proteins identified by analyses on an LQT-Orbitrap mass spectrometer (Thermo), as described^21^. A detailed protocol for the ChIP-MS procedure can be found at http://www.nature.com/protocolexchange/. For western blot analyses, ChIP samples were separated on a 4–12% polyacrylamide gel (Novex) and nitrocellulose blots probed with antibodies against the used histone modifications (see above, 1:500 dilution, pan histone H3 antibody (Abcam 1791, 1:1,000 dilution) and Nanog (Cosmo Bio Ltd., 1:2,000 dilution). Non-cropped versions of the western blot panels in Fig. 1d can be found in Supplementary Fig. 3.

### ChIP-MS inclusion and prediction criteria

Two independent ChIPs were performed for each tested histone modification and for GFP, as control ChIPs, and analysed by MS. For inclusion into the ChIP-MS list of identified proteins (Supplementary Tables 1 and 2), factors needed to be identified by MS with a Mascot score of 50 or higher in at least one histone modification ChIP. A Mascot score of at least 45 in any of the other ChIPs was annotated in the ChIP-MS list. In case of Mascot scores between 45 and 60, individual peptide MS/MS spectra were checked manually and interpreted as valid identifications or discarded. In addition, the Mascot programme was used to determine the Mascot peptide significance threshold (*P*<0.05) in the ChIP-MS samples. Significance thresholds were H3K4me3 (Experiment 1); Mascot score 28, H3K4me3 (Experiment 2); Mascot score 28, H3K4me1 (Experiment 1); Mascot score 28, H3K4me1 (Experiment 2); Mascot score 28, H3K27ac (Experiment 1); Mascot score 28, H3K27ac (Experiment 2); Mascot score 28, H3K9me3 (Experiment 1); Mascot score 28, H3K9me3 (Experiment 2); Mascot score 29, GFP (Experiment 1); Mascot score 28, GFP (Experiment 2); Mascot score 28. For inclusion into the ChIP-MS list (Supplementary Tables 1 and 2), protein identifications had to be based on peptides with a Mascot score at or above the Mascot peptide significance threshold of the sample in which the peptides were observed. For assessment of the quantity of the identified proteins in the ChIP-MS samples, we used emPAI a calculation method based on the number of peptide spectra identified by MS, normalized for the number of peptides that theoretically should be identifiable for that protein^22^ (to compensate for large proteins, which likely have more MS peptides). Inclusion into the ChIP-MS list required an at least fivefold higher average emPAI score in the ChIPs for at least one histone modification compared with any of the anti-GFP control ChIPs. Inclusion into the ChIP-MS list further required a factor to have an at least threefold higher average emPAI score in the two ChIPs for one histone modification compared with the two ChIPs for one or more of the other histone modifications, to exclude factors that bind chromatin indiscriminately of the tested histone modifications. Cytoskeletal and cytoplasmic proteins were excluded. Average emPAI scores were calculated from the two independent ChIP-MS experiments. Localization prediction was according to the following criteria; highest average emPAI score in H3K4me3 ChIP samples gives ‘promoter' prediction, highest average emPAI score in the H3K4me1 ChIP samples gives ‘enhancer' prediction, highest average emPAI score in the H3K9me3 ChIP samples gives ‘heterochromatin' prediction. In case, average emPAI scores for the H3K4me3 and H3K4me1 ChIP samples were equal, the prediction was ‘promoter'. The H3K27ac ratio of a factor is defined as the ratio of its average H3K27ac emPAI score over the average H3K4me3 emPAI score or average H3K4me1 emPAI score, whichever one is the highest.

### ChIP and sequencing

Anti-V5 ChIPs were performed as described^21^. For V5-Dppa2 ChIP, CGR8 ESCs stably expressing V5-Dppa2 (see above) were used, for the control ChIP, the parental CGR8 parental ESC line was used. For each ChIP, 100 × 10^6^ ESCs were used. Precipitated DNA was analysed by quantitative PCR or used for library generation followed by next-generation sequencing on an Illumina Genome analyser, as described^20^.

### Data analysis

Sequences with low complexity that are unlikely to map uniquely to the genome were removed from the Dppa2 ChIP-seq, modified ChIP-seq experiments for the used histone modifications and published ChIP-seq data sets (Supplementary Table 5), using prinseq-lite with the dust method with 7 as threshold^44^. The remaining sequences with a Phred score <70 were mapped to the mm9 reference genome using Bowtie^45^ v0.12.7, where we used a seed length of 36 in which we allowed a maximum of two mismatches. If a read had multiple alignments only the best matching read was reported. Duplicated reads were removed. MACS^46^ v1.4.2 was used for peak calling of Esrrb, Nanog, Oct4, Polr2a, P300, H3K4me1 and H3K4me3 using default settings. ChIP-seq data sets with multiple replicates were merged. For peak calling the Polr2a, P300, H3K4me1, H3K4me3 ChIP-seq data sets, the sequenced input was used as control. For peak calling Esrrb, Nanog and Oct4, the GFP ChIP was used as a control (Supplementary Table 5). For Dppa2, peak calling, we provided MACS1.4.2 with a shift size of 75 base pairs. Peaks with a *P*-value≤1 × 10^−10^ were retained for Dppa2, Oct4, Nanog, Polr2a, H3K4me3, H3K4me1 and sites with a *P*-value≤2 × 10^−10^ for Esrrb and P300. For Dppa2, only peaks with at least 100 aligned reads were retained. Dppa2, Oct4, Nanog and Esrrb peaks were considered to overlap if their peak summit was within 125 base pairs of each other. Venn diagrams, violin plots and bubble plots were created in R using the VennDiagram, vioplot and ggplots2 packages, respectively. To calculate the overlap between the mapped reads from the modified ChIP-seq experiments for H3K4me3, H3K4me1, H3K27ac and promoters or enhancers. Promoters were defined as the regions from −1 to +1 kb of the summits of a significant RNAPol2-binding sites (Polr2a) within 1 kb of a transcription start site (TSS). Enhancers were defined as the regions from −1 to +1 kb of the summits of a significant P300-binding sites that were not within 1 kb of a TSS. The sequencing profiles of the conventional ChIP and modified ChIP for the used histone modifications and the Dppa2 ChIP-seq experiments (Figs 1g and 4c and Supplementary Fig. 1) were created in the IGV browser^47^.

### Genome-wide correlation

To calculate the genome-wide correlation between the published histone modification ChIP-seq data sets and the protein factors, we divided the entire genome in bins of 1,000 base pairs and calculated reads per million (RPM) for all bins in all data sets. The input was subtracted. For each histone modification and protein factor, we selected the 4,000 bins with the highest RPM. A unified list was created for each individual protein factor, containing the selected bins of the four used histone modifications and that of the protein factor itself. The Spearman correlation coefficients of the protein factor with the different histone modifications were calculated from this list. To calculate the correlations between conventional and modified ChIP-seq experiments for the used histone modifications, we used a unified list that included the 4,000 bins with the highest RPM for each conventional and modified ChIP-seq experiment.

### Heatmaps

To assign promoter regions, H3K4me3 peak summits, as determined by MACS (see above) were required to be within 1 kb range of a TSS, resulting in 12,913 promoter regions. To assign enhancer regions, H3K4me1 peak summits were filtered against the presence of H3K4me3 signal in a region from −4.1 and+4.1 kb around the H3K4me1 peak summit, resulting in 30,564 enhancer regions. Promoters were sorted for number of H3K27ac reads present in the central 2 kb of the promoter region. Enhancers were sorted for the number of H3K27ac reads in the central 8.2 kb of the enhancer region, to also include broad enhancers. For both promoters and enhancers, we displayed a region from −4.1 to +4.1 kb around the peak summits, divided into 51 bins of 160 bp each. Promoter and enhancer heatmaps for each protein factor or histone modification were normalized by calculating the RPM based on the sum of all reads found in the displayed promoter and enhancer region for that factor.

### Expression of Dppa2 and Oct4 target genes

Dppa2-bound genes contained a MACS-called Dppa2 peak (see above) within 1 kb from the TSS. Dppa2 target genes were defined as Dppa2-bound genes with at least twofold difference in expression in Dppa2 knockout ESCs, compared with wild-type ESCs and an adjusted *P*-value of ≤0.10, in a Dppa2 knockout microarray data set^31^. The GEO2R script, as provided by the authors on the Gene Expression Omnibus (GEO), was used to calculate fold change in expression and adjusted *P*-value for each probe. Multiple probes to the same gene were aggregated by taking the average fold change. Oct4-bound genes contained a MACS-called Oct4 peak (see above) within 20 kb from the TSS. Oct4 target genes were defined as Oct4-bound genes with at least twofold difference in expression between 24 and 0 h after Oct4 knockdown^48^ in ZHBTc4 ESCs that have their only intact Oct4 gene under doxycycline control. The used microarray data set^48^ was already normalized by the authors. A published RNA sequencing data set consisting of two replicates^49^ was used to calculate the mean expression of Dppa2- or Oct4-bound genes and Dppa2- or Oct4-target genes. Both replicates were mapped against mouse reference NCBIM37.67 using Tophat^50^ v2.0.11 with default settings and a segment length of 20. The aligned exon reads were counted and normalized using Bioconductor DESeq2 package in R. Replicates were normalized by dividing the counts by their size factors. The expression level per gene was calculated by taking the average of both replicates and calculating the reads per kb for each gene. To calculate the fold change of Dppa2- and Oct4-target genes in differentiated tissues over ESCs, we used the BioGPS mouse MOE430 Gene Atlas^51^. The same database was used to determine the tissue or cell line in which the Dppa2 and Oct4 target genes were highest expressed.

## Additional information

**Accession codes:** ChIP sequencing data are available through the Gene Expression Omnibus (NCBI), accession code GSE58113.

**How to cite this article:** Engelen, E. *et al*. Proteins that bind regulatory regions identified by histone modification chromatin immunoprecipitations and mass spectrometry. *Nat. Commun.* 6:7155 doi: 10.1038/ncomms8155 (2015).

## Supplementary Material

Supplementary Information Supplementary Figures 1-3, Supplementary Tables 1-5 and Supplementary References.

Supplementary Dataset Dppa2-bound transcription start sites in mouse embryonic stem cells
