# Supplementary material for: Proteins that bind regulatory regions identified by histone modification chromatin immunoprecipitations and mass spectrometry
Source: Nat Commun. 2015 May 20;6:7155. doi: 10.1038/ncomms8155 (PMC4455091; doi:10.1038/ncomms8155)
Supplement: Supplementary Information — Supplementary Figures 1-3, Supplementary Tables 1-5 and Supplementary References. [file ncomms8155-s1.pdf]

Supplementary information

Proteins that bind regulatory regions identified by histone-modification chromatin immunoprecipitations and mass spectrometry

Erik Engelen[1*], Johannes H. Brandsma[1*], Maaike J. Moen[1], Luca Signorile[1], Dick H.W. Dekkers[2], Jeroen Demmers[2], Christel E.M. Kockx[3], Zehila Ozgür[3], Wilfred F. J. van IJcken[3], Debbie L.C. van den Berg[1,4], Raymond A. Poot[1]

[1] Department of Cell Biology, Erasmus MC, Wytemaweg 80, 3015 CN Rotterdam, The Netherlands

[2] Center for Proteomics, Erasmus MC, The Netherlands

[3] Center for Biomics, Erasmus MC, The Netherlands

[4] The Francis Crick Institute, Mill Hill Laboratory, The Ridgeway, London NW7 1AA, United Kingdom

* These authors contributed equally to this work

Correspondence to:

Raymond A. Poot

r.poot@erasmusmc.nl

Phone: +31-10-7043352

Fax: +31-10-7044743

Debbie L.C. van den Berg

debbie.vandenberg@crick.ac.uk

Phone: +44-20-88162574

Fax: + 44-20-89064477

Table of contents

Supplementary Figures

Supplementary Figure 1: Modified ChIP-seq tracks around pluripotency gene Nanog

Supplementary Figure 2: Positions of Oct4 binding sites on Oct4 target genes

Supplementary Figure 3: Western Blots (full scans) for ChIP-MS samples (as in Figure 1d)

Supplementary Tables

Supplementary Table 1: ChIP-MS identified proteins, predictions and phenotypes

Supplementary Table 2: ChIP-MS identified proteins

Supplementary Table 3: ChIP-MS identified complex subunits and predictions

Supplementary Table 4: Dppa2 target genes

Supplementary Table 5: Accession numbers of studies used for genome-wide correlations

References for pluripotency phenotypes of ChIP-MS identified factors (Supplementary Table 1)

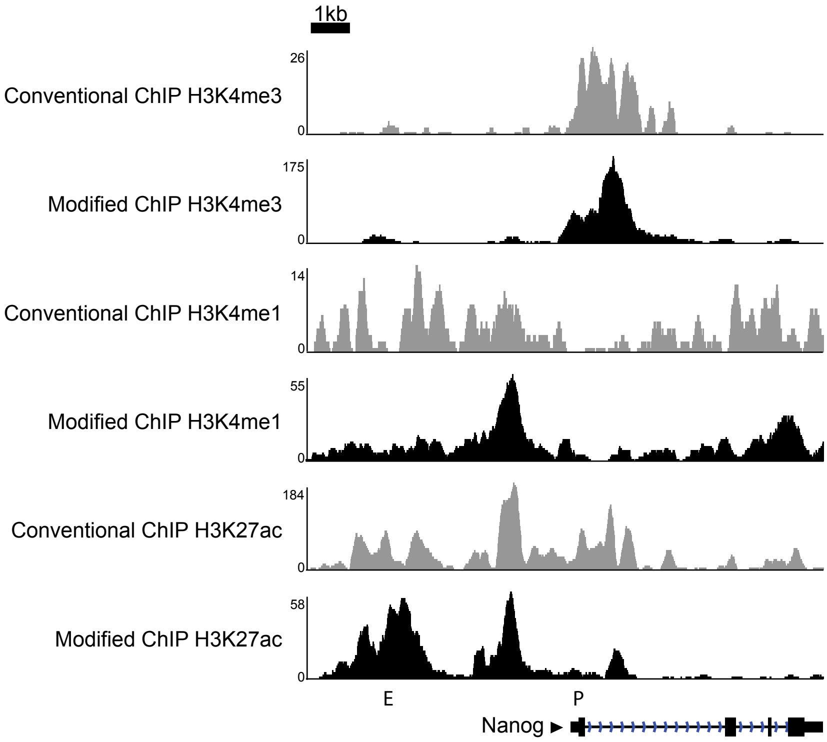

Supplementary Figure 1. Modified ChIP-seq tracks around pluripotency gene *Nanog*.

ChIP-seq tracks for modified ChIP or conventional ChIP for H3K4me3, H3K4me1 or H3K27ac around pluripotency gene *Nanog*. Sequence reads were plotted relative to chromosomal position. Genome location of *Nanog* is shown, scale bar indicates 1 kb of genome. P indicates promoter, E indicates putative enhancer.

a

b

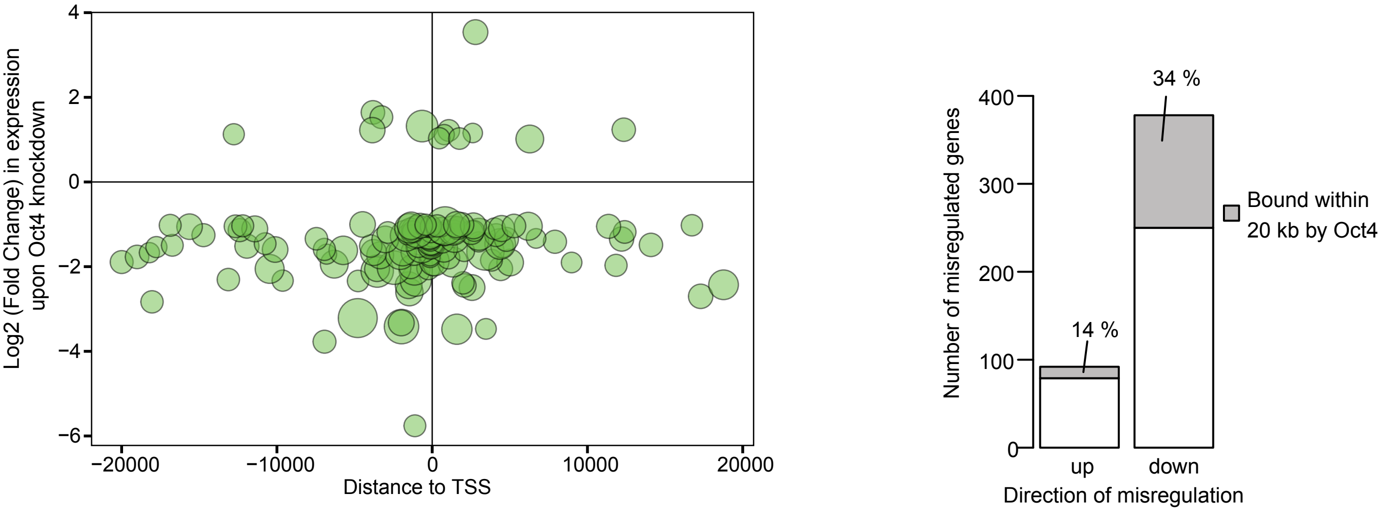

Supplementary Figure 2. Position of Oct4 binding sites on Oct4 target genes.

(**a**) Bubble plot indicating the positions of Oct4 binding sites relative to the transcription start site (TSS) on genes that are either up-regulated ≥ 2 fold (upper part) or down-regulated ≥ 2 fold (lower part) after 24 h of Oct4 depletion in mouse ESCs. Log2 of the fold change in expression is indicated on the Y-axis, Oct4 binding distance from the TSS (in base pairs) is indicated on the X-axis. Size of the bubbles correlates with fold difference of Oct4 ChIP peak over control.

(**b**) Bar diagram showing the total number of up regulated and down regulated genes upon Oct4 depletion and the number of these genes bound by Oct4 within 20 kb from the TSS (grey areas). The number of Oct4-bound genes is also indicated as a percentage of the total number of up-regulated or down-regulated genes.

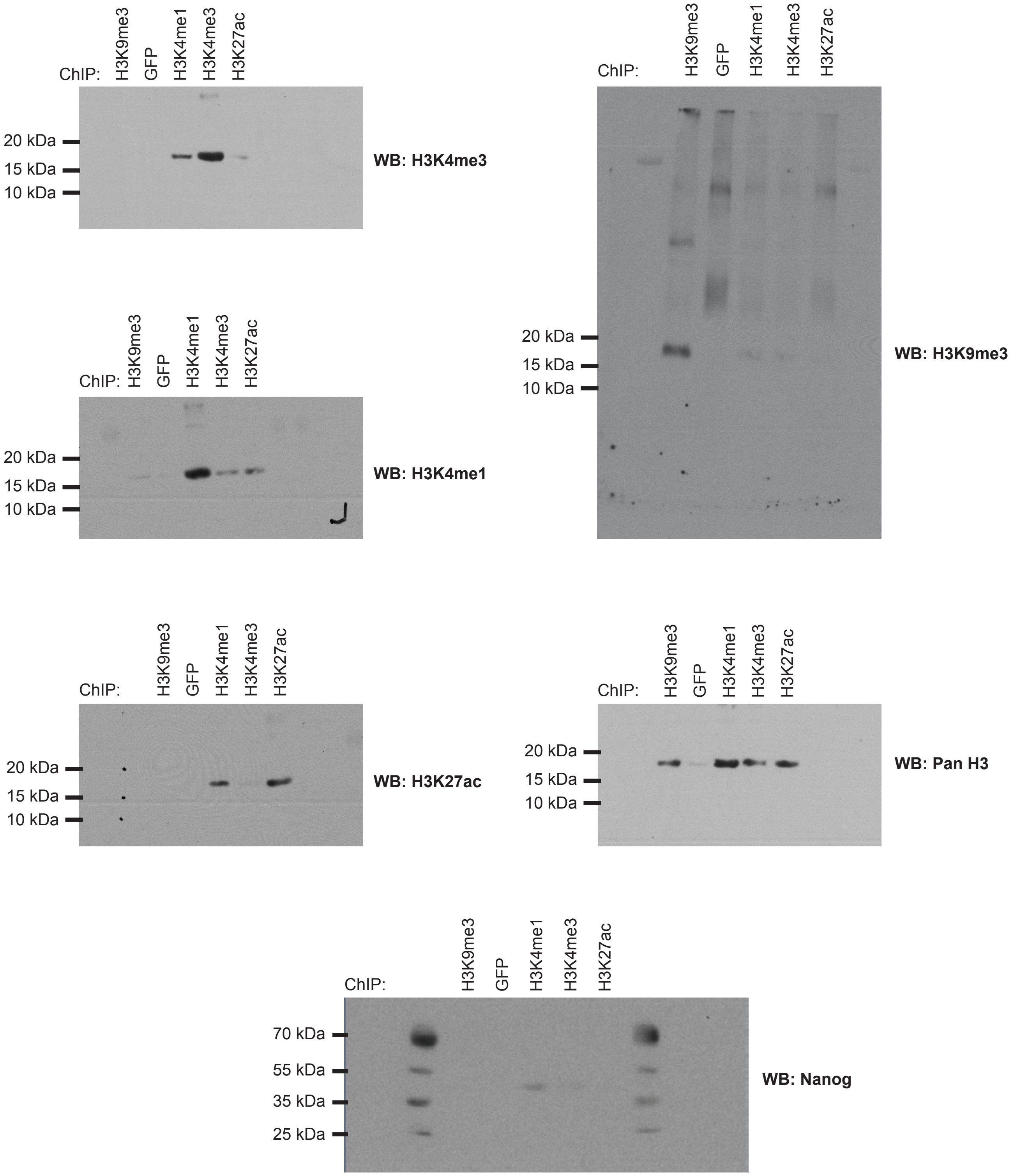

Supplementary Figure 3. Western Blots (full scans) for ChIP-MS samples (as in Figure 1d).

Western blots for histone modifications and Nanog for ChIP-MS samples. ChIP-MS samples (top), molecular weight markers (left) and antibody used for Western blot (right) are indicated.

Supplementary Table 1. ChIP-MS identified proteins, predictions and phenotypes

Protein	Complex[a]	H3K4me3 avrg emPAI	H3K27ac avrg emPAI	H3K4me1 avrg emPAI	H3K9me3 avrg emPAI	GFP avrg emPAI	H3K27ac ratio	Prediction[b]	Pluripotency phenotype[c]
Actl6a	BAF + INO80 + Trrap/Ep400	0,29	0,16	0,12	0,08		0,55	Promoter	-
Actr3	Arp2/3	0,04					0	Promoter	-
Actr5			0,03					Unclear	-
Adnp				0,03	0,02		0	Enhancer	-
Ankhd1				0,10			0	Enhancer	-
Anln					0,03			Heterochromatin	-
Anp32a	SET	0,17		0,18			0	Enhancer	-
Arid1a	BAF		0,07	0,06			1,17	Enhancer	yes
Arid4a	Sin3	0,12					0	Promoter	-
Ash2l	MLL	0,25					0	Promoter	yes
Atad2b			0,03	0,04	0,04		0,75	Enhancer	-
Atrx					0,09			Heterochromatin	-
Aurkb	Aurora K	0,22	0,10	0,10	0,65			Heterochromatin	yes
Banf1		0,66	0,47	0,63			0,71	Promoter	yes
Bap18	MLL	0,63					0	Promoter	-
Baz1a	ACF			0,01			0	Enhancer	-
Baz2a	NoRC	0,06	0,22	0,20	0,07		1,10	Enhancer	-
Bend3		0,24		0,04			0	Promoter	-
Bptf	NURF	0,08	0,02	0,02			0,25	Promoter	yes
Brd1	MOZ/MORF	0,03	0,05	0,10			0,50	Enhancer	-
Brd2		0,37	0,75	0,18			2,03	Promoter	-
Brd3		0,12	0,32				2,67	Promoter	-
Brd4		0,03	0,21	0,10			2,10	Enhancer	-
Brms1	Sin3	0,14					0	Promoter	-
Brms1l	Sin3	0,47					0	Promoter	-
Brpf1	MOZ/MORF	0,16					0	Promoter	-
Brpf3	MOZ/MORF	0,03					0	Promoter	-
Cad				0,04			0	Enhancer	-
Cald1		0,06					0	Promoter	-
Cbx7	PRC1	0,10		0,10			0	Promoter	yes
Cdc5l	Prp19			0,04			0	Enhancer	-
Cdc73	PAF1	0,10	0,10				1,00	Promoter	yes
Cdca7			0,09	0,09			1,00	Enhancer	-
Cdca8	Aurora K	0,28	0,13	0,24	1,17			Heterochromatin	-
Cdk7	TFIIH	0,05					0	Promoter	-
Cdk9	P-TEFb		0,09					Unclear	yes
Cep95			0,02	0,04			0,50	Enhancer	-
Chaf1b	CAF1		0,23	0,52			0,44	Enhancer	yes
Chd1		0,26	0,24	0,14	0,02		0,92	Promoter	yes
Chd2		0,05		0,03			0	Promoter	-
Chd8		0,15	0,03				0,20	Promoter	-
Ckb		0,14					0	Promoter	-
Coil					0,03			Heterochromatin	-
Cpsf6	CFIm	0,07	0,07				1,00	Promoter	-
Creb1		0,11					0	Promoter	-
Cse1l		0,04					0	Promoter	-
Csnk2a1		0,89	0,27	0,21			0,30	Promoter	-
Csnk2b		0,82	0,26	0,08			0,32	Promoter	-
Csrp2bp	ATAC	0,04					0	Promoter	-

Protein	Complex[a]	H3K4me3 avrg emPAI	H3K27ac avrg emPAI	H3K4me1 avrg emPAI	H3K9me3 avrg emPAI	GFP avrg emPAI	H3K27ac ratio	Prediction[b]	Pluripotency phenotype[c]
Ctbp2		0,46	0,30	0,43	0,04		0,65	Promoter	yes
Ctcf		0,07		0,05			0	Promoter	-
Ctr9	PAF1	0,03	0,03				1,00	Promoter	yes
Cul4b	DDB1/Cul4 ubiquitin ligase	0,17	0,18	0,18			1,00	Enhancer	-
Ddb1	DDB1/Cul4 ubiquitin ligase	0,19	0,24	0,29			0,83	Enhancer	-
Ddx47					0,15			Heterochromatin	-
Ddx54				0,04			0	Enhancer	-
Dido1		0,02					0	Promoter	-
Dis3			0,05	0,05			1,00	Enhancer	-
Dmap1	Trrap/Ep400	0,07		0,04			0	Promoter	yes
Dnajc9		0,07	0,14	0,14			1,00	Enhancer	-
Dpf2	BAF		0,22	0,12			1,83	Enhancer	-
Dppa2		0,61	0,06	0,29			0,10	Promoter	yes
Dppa4		0,58		0,39			0	Promoter	yes
Dpy30	MLL	0,18					0	Promoter	yes
Dut		0,41	0,18	0,18			0,44	Promoter	-
Emsy		0,02					0	Promoter	-
Ep300			0,05					Unclear	yes
Ep400	Trrap/Ep400		0,02					Unclear	yes
Erh		0,37					0	Promoter	-
Esrrb			0,08	0,20			0,40	Enhancer	yes
Ezh2	PRC2	0,07	0,04	0,09			0,44	Enhancer	yes
Fam60a	Sin3	0,24					0	Promoter	-
Fbxl19	SCF ubiquitin ligase	0,05					0	Promoter	-
Fkbp4				0,07			0	Enhancer	-
Gltscr2				0,07			0	Enhancer	-
Glyr1		0,19	0,24	0,26	0,07		0,92	Enhancer	-
Gm53		0,19					0	Promoter	-
Gtf2e1	TFIIE			0,08			0	Enhancer	-
Gtf2i			0,04	0,07			0,57	Enhancer	-
Gtf3c1	TFIIIC	0,01	0,01	0,02			0,50	Enhancer	-
Hat1		0,11	0,04	0,04			0,36	Promoter	-
Hcfc1	MLL	0,05					0	Promoter	yes
Hdac1	NuRD + Sin3 + REST	1,02	0,30	0,38	0,11		0,29	Promoter	yes
Hdac2	NuRD + Sin3 + REST	0,40		0,10			0	Promoter	-
Hdgfrp2			0,05					Unclear	-
Hmga2		0,14	0,14		0,28			Heterochromatin	-
Hmgb3		0,11		0,1			0	Promoter	-
Hmgxb4		0,20					0	Promoter	-
Hp1bp3		0,26		0,38	0,23		0	Enhancer	-
Incenp	Aurora K	0,06			0,26			Heterochromatin	-
Ing1	Sin3	0,06					0	Promoter	-
Ing2	Sin3	0,11					0	Promoter	-
Ing3	Trrap/Ep400	0,04	0,04				1,00	Promoter	-
Ing4	Trrap/Ep400	0,14		0,07			0	Promoter	-
Ing5	MOZ/MORF	0,58	0,13	0,20			0,22	Promoter	yes
Ino80	INO80	0,05	0,05				1,00	Promoter	yes
Ints1	Integrator			0,02			0	Enhancer	-
Irgc				0,04			0	Enhancer	-
Jarid2	PRC2	0,16	0,05	0,21	0,04		0,24	Enhancer	yes
Kdm1a	BHC	0,06	0,02	0,02	0,02		0,33	Promoter	yes
Kdm2a	SCF ubiquitin ligase	0,16	0,03	0,03			0,19	Promoter	-

Protein	Complex[a]	H3K4me3 avrg emPAI	H3K27ac avrg emPAI	H3K4me1 avrg emPAI	H3K9me3 avrg emPAI	GFP avrg emPAI	H3K27ac ratio	Prediction[b]	Pluripotency phenotype[c]
Kdm2b	PRC1	0,09		0,01			0	Promoter	yes
Kdm4a		0,03					0	Promoter	-
Kdm4c		0,65	0,03	0,13			0,05	Promoter	yes
Kdm5a		0,02					0	Promoter	-
Kdm5b		0,25	0,02	0,12			0,08	Promoter	yes
Kif2c				0,05			0	Enhancer	-
Kif4				0,03			0	Enhancer	-
Kifc5b			0,07	0,15			0,47	Enhancer	-
Klf16		0,16					0	Promoter	-
Klf5			0,07	0,04			1,75	Enhancer	yes
L3mbtl2		0,08		0,07			0	Promoter	yes
Las1l	5FMC			0,06			0	Enhancer	-
Lasp1		0,29		0,13			0	Promoter	-
Lig1		0,08		0,02			0	Promoter	-
Lig3			0,02	0,05			0,40	Enhancer	-
Llph				0,13			0	Enhancer	-
Lmna		0,13	0,08	0,08	0,48			Heterochromatin	-
Lmnb1		0,99	1,60	1,83	3,15	0,37		Heterochromatin	-
Lmnb2		0,12	0,24	0,30	0,98			Heterochromatin	-
Lrwd1	ORC	0,14		0,11	0,35			Heterochromatin	-
Lsm2	U6 SnRNP		0,18					Unclear	-
Luc7l3				0,04			0	Enhancer	-
Max		0,11	0,11	0,10			1,00	Promoter	yes
Mbtd1		0,06					0	Promoter	-
Mdc1			0,03	0,05			0,60	Enhancer	-
Meaf6	Trrap/Ep400	0,51	0,19	0,19			0,37	Promoter	-
Men1	MLL	0,32	0,03	0,15			0,09	Promoter	-
Mll2	MLL	0,21		0,02		0,01	0,00	Promoter	-
Morc2a			0,05					Unclear	-
Morc3		0,70	0,24	0,24	0,02		0,34	Promoter	-
Mta3	NuRD	0,24	0,14	0,27			0,52	Enhancer	-
Mtf2	PRC2	0,16	0,06	0,22	0,06		0,27	Enhancer	yes
Mycn		0,08	0,08				1,00	Promoter	yes
Myst2	HBO1	0,56	0,36	0,39	0,06		0,64	Promoter	yes
Myst3	MOZ	0,08					0	Promoter	yes
Myst4	MORF	0,04					0	Promoter	-
Nacc1		0,04	0,07	0,13			0,54	Enhancer	yes
Nasp			0,02	0,02			1,00	Enhancer	-
Nfrkb			0,03					Unclear	yes
Nolc1				0,05			0	Enhancer	-
Nrf1		0,08					0	Promoter	-
Nsd1		0,01	0,04	0,05			0,80	Enhancer	-
Nudt13				0,10			0	Enhancer	-
Nudt21	CFIm	0,48					0	Promoter	-
Numa1		0,22	0,15	0,29	0,10		0,52	Enhancer	-
Oct4		0,16	0,10	0,21	0,05		0,48	Enhancer	yes
Ogt		0,15	0,02	0,05			0,13	Promoter	yes
Pak1ip1		0,19					0	Promoter	-
Parp2				0,03			0	Enhancer	-
Parp9					0,04			Heterochromatin	-
Patz1		0,05		0,05			0	Promoter	-
Pbrm1	BAF	0,02	0,11	0,11			1,00	Enhancer	-
Pds5a	Wapl		0,03	0,05			0,60	Enhancer	-

Protein	Complex[a]	H3K4me3 avrg emPAI	H3K27ac avrg emPAI	H3K4me1 avrg emPAI	H3K9me3 avrg emPAI	GFP avrg emPAI	H3K27ac ratio	Prediction[b]	Pluripotency phenotype[c]
Pds5b	Wapl	0,02	0,01	0,02			0,50	Enhancer	-
Phc1	PRC1			0,04			0	Enhancer	-
Phf16	HBO1	0,04					0	Promoter	-
Phf20				0,02			0	Enhancer	yes
Phf23		0,53		0,09			0	Promoter	yes
Phf8		0,23	0,02				0,09	Promoter	-
Phip		0,04	0,07	0,13			0,54	Enhancer	-
Pin1			0,11	0,11			1,00	Enhancer	yes
Pnp		0,20					0	Promoter	-
Polr1c	Pol I	0,3					0	Promoter	-
Polr2a	Pol II	0,15	0,13	0,06			0,87	Promoter	-
Polr2b	Pol II	0,43	0,33	0,07			0,77	Promoter	-
Polr2c	Pol II	0,57	0,16	0,11			0,28	Promoter	-
Polr2e	Pol II	0,85	0,24	0,08			0,28	Promoter	-
Polr2g	Pol II	0,22					0	Promoter	-
Ppp2cb				0,11			0	Enhancer	-
Prdm10		0,03	0,03				1,00	Promoter	-
Prdm2				0,02			0	Enhancer	-
Prrc2c				0,01			0	Enhancer	-
Psma5	Proteasome	0,07		0,07			0	Promoter	-
Ptcd3				0,12			0	Enhancer	-
Qser1		0,03		0,01			0	Promoter	-
Rad21	Cohesin		0,03	0,03			1,00	Enhancer	yes
Rars		0,05					0	Promoter	-
Rbbp4	NuRD/CAF1	1,51	0,62	1,09	0,37	0,12	0,41	Promoter	-
Rbbp5	MLL	0,14					0	Promoter	yes
Rbl1				0,03			0	Enhancer	-
Rbpj		0,30		0,20	0,14		0	Promoter	-
Rfc3		0,05	0,10				2,00	Promoter	-
Rfc4		0,20	0,15	0,19			0,75	Promoter	-
Rnf2	PRC1	0,35		0,30			0	Promoter	yes
Rsf1	RSF	0,05	0,11	0,12			0,92	Enhancer	-
Ruvbl2	Trrap/Ep400	1,36	1,91	0,41	0,16		1,40	Promoter	yes
Samd1		0,26					0	Promoter	-
Sap130	Sin3	0,18					0	Promoter	-
Sap30	Sin3	0,29					0	Promoter	-
Sap30l	Sin3	0,09					0	Promoter	-
Sbno1				0,01			0	Enhancer	-
Sephs1		0,14	0,05				0,36	Promoter	-
Sf1		0,14					0	Promoter	-
Sf3b1				0,04			0	Enhancer	-
Shprh		0,04	0,01	0,03			0,25	Promoter	-
Sin3a	Sin3	1,34	0,15	0,30			0,11	Promoter	yes
Sin3b	Sin3	0,09					0	Promoter	-
Skp1a	SCF ubiquitin ligase	0,45	0,23	0,10			0,51	Promoter	-
Smarca4	BAF	0,14	0,28	0,22	0,03		1,27	Enhancer	yes
Smarcb1	BAF		0,09	0,05			1,80	Enhancer	yes
Smarcc1	BAF	0,10	0,46	0,31	0,07		1,48	Enhancer	yes
Smarcd1	BAF		0,50	0,35			1,43	Enhancer	-
Smc1a	Cohesin	0,08	0,12	0,22	0,06		0,55	Enhancer	yes
Smc6	Smc5/Smc6			0,03			0	Enhancer	yes
Smchd1			0,02	0,03	0,04			Heterochromatin	-
Spin1		0,72	0,13	0,13			0,18	Promoter	-

Protein	Complex[a]	H3K4me3 avrg emPAI	H3K27ac avrg emPAI	H3K4me1 avrg emPAI	H3K9me3 avrg emPAI	GFP avrg emPAI	H3K27ac ratio	Prediction[b]	Pluripotency phenotype[c]
Srrt		0,04	0,04	0,08			0,50	Enhancer	-
Ssrp1	FACT	0,68	0,93	0,73	0,29	0,13	1,27	Enhancer	-
Suds3	Sin3	0,41					0	Promoter	-
Supt4h2	DSIF	0,34					0	Promoter	yes
Supt5h	DSIF	0,22	0,16	0,06			0,73	Promoter	-
Supt6h		0,07	0,09				1,29	Promoter	-
Suv39h2					0,31			Heterochromatin	yes
Suz12	PRC2	0,31	0,12	0,40	0,07		0,30	Enhancer	yes
Taf1	TFIID	0,03					0	Promoter	yes
Taf2	TFIID	0,06	0,02				0,33	Promoter	yes
Taf3	TFIID	0,02	0,02				1,00	Promoter	yes
Taf4a	TFIID	0,11					0	Promoter	yes
Taf5	TFIID	0,05					0	Promoter	yes
Taf6	TFIID	0,20	0,14				0,70	Promoter	yes
Taf7	TFIID	0,16	0,11				0,69	Promoter	yes
Taldo1			0,05	0,11			0,45	Enhancer	-
Tbrg4				0,09			0	Enhancer	-
Tcea1	TFIIS	0,12	0,26	0,05			2,17	Promoter	-
Tcerg1		0,02	0,03	0,02			1,50	Enhancer	-
Tcof1					0,02			Heterochromatin	-
Tead1			0,04	0,04			1,00	Enhancer	-
Tlk2				0,06			0	Enhancer	-
Trim24			0,25	0,04			6,25	Enhancer	-
Trim33			0,08					Unclear	-
Trp53			0,06					Unclear	-
Trrap	Trrap/Ep400	0,05	0,05	0,01			1,00	Promoter	yes
Uba1			0,03	0,03			1,00	Enhancer	-
Ube2h				0,24			0	Enhancer	-
Ubtf		0,79	0,44	0,43			0,56	Promoter	-
Usp48		0,15	0,08	0,21	0,05		0,38	Enhancer	-
Utp14b				0,07			0	Enhancer	-
Vmn2r100		0,02		0,06			0	Enhancer	-
Wdr18	5FMC	0,04		0,12	0,08		0	Enhancer	-
Wdr5	MLL	0,60	0,27	0,17			0,45	Promoter	yes
Wdr55		0,05					0	Promoter	-
Zfp280c		0,05			0,22			Heterochromatin	-
Zfp281		0,04					0	Promoter	yes
Zfp462		0,01			0,02			Heterochromatin	-
Zic5		0,03					0	Promoter	-
Zmynd8	Integrator		0,05	0,04			1,25	Enhancer	-
Znf512		0,13		0,24	0,25			Heterochromatin	-
Zscan10		0,04		0,09			0	Enhancer	yes

[a] Subunit of indicated protein complex

[b] Prediction of genome localization based on our ChIP-MS criteria

[c] ESC pluripotency phenotype (references in supplementary material)

Engelen, Brandsma, Supplementary Table 1

Supplementary Table 2. ChIP-MS identified Proteins

	H3K4me3								H3K27ac								H3K4me1								H3K9me3								GFP							
	Experiment 1				Experiment 2				Experiment 1				Experiment 2				Experiment 1				Experiment 2				Experiment 1				Experiment 2				Experiment 1				Experiment 2			
Protein	Mascot	emPAI	Unique pept.[a]	Pept. count[b]	Mascot	emPAI	Unique pept.[a]	Pept. count[b]	Mascot	emPAI	Unique pept.[a]	Pept. count[b]	Mascot	emPAI	Unique pept.[a]	Pept. count[b]	Mascot	emPAI	Unique pept.[a]	Pept. count[b]	Mascot	emPAI	Unique pept.[a]	Pept. count[b]	Mascot	emPAI	Unique pept.[a]	Pept. count[b]	Mascot	emPAI	Unique pept.[a]	Pept. count[b]	Mascot	emPAI	Unique pept.[a]	Pept. count[b]	Mascot	emPAI	Unique pept.[a]	Pept. count[b]
Actl6a	305	0.57	6	25					122	0.16	2	9	81	0.16	2	5	191	0.24	3	9					91	0.16	2	4												
Actr3	56	0.08	1	2																																				
Actr5													79	0.05	1	1																								
Adnp																	97	0.06	3	3					66	0.03	1	1												
Ankhd1																	111	0.19	3	5																				
Anln																													63	0.06	2	2								
Anp32a	75	0.17	2	3	47	0.17	1	2													74	0.36	2	3																
Arid1a									170	0.05	4	4	235	0.08	5	5	202	0.07	5	6	241	0.05	3	3																
Arid4a	79	0.24	2	4																																				
Ash2l	195	0.35	5	15	77	0.14	2	3																																
Atad2b									89	0.06	2	2					101	0.04	2	2	80	0.04	2	2					152	0.07	3	4								
Atrx																									259	0.1	7	11	264	0.07	6	7								
Aurkb					238	0.44	4	18					69	0.2	2	2	70	0.19	2	4					276	0.72	6	14	252	0.57	5	17								
Banf1	107	0.93	2	6	59	0.39	1	2	78	0.93	2	2					103	0.87	2	5	71	0.39	1	3																
Bap18	166	0.79	3	30	119	0.47	2	8																																
Baz1a																	55	0.02	1	2																				
Baz2a	57	0.04	2	2	239	0.07	5	7	363	0.21	10	19	579	0.23	11	23	526	0.21	12	20	468	0.19	9	24	191	0.09	5	8	110	0.04	2	7								
Bend3	196	0.26	5	7	206	0.21	5	5													77	0.08	2	2																
Bptf	422	0.11	10	17	173	0.04	4	10	102	0.03	3	3					114	0.02	2	3	115	0.02	2	2																
Brd1	70	0.06	2	5					57	0.03	1	2	88	0.06	2	2	156	0.08	3	7	142	0.11	4	9																
Brd2	471	0.51	10	45	273	0.23	5	11	527	0.57	11	30	750	0.93	14	53	191	0.17	4	6	231	0.18	4	15																
Brd3	119	0.15	3	5	81	0.09	2	4	278	0.44	7	14	174	0.2	4	11																								
Brd4	72	0.05	2	5					313	0.23	9	15	323	0.18	6	33					132	0.19	3	13																
Brms1	81	0.28	2	2																																				
Brms1l	179	0.6	5	5	177	0.33	3	5																																

	H3K4me3							
	Experiment 1				Experiment 2			
Protein	Mascot	emPAI	Unique pept.[a]	Pept. count[b]	Mascot	emPAI	Unique pept.[a]	Pept. count[b]
Brpf1	233	0.23	7	11	91	0.08	3	3
Brpf3					86	0.05	2	5
Cad								
Cald1	61	0.12	2	2				
Cbx7	50	0.19	1	4				
Cdc5l								
Cdc73	82	0.2	3	3				
Cdca7								
Cdca8					191	0.56	4	20
Cdk7					51	0.1	1	1
Cdk9								
Cep95								
Chaf1b								
Chd1	672	0.34	15	48	382	0.18	8	29
Chd2	177	0.09	4	22				
Chd8	516	0.23	14	33	199	0.07	4	12
Ckb	66	0.18	2	4	60	0.09	1	1
Coil								
Cpsf6					88	0.13	2	13
Creb1	60	0.22	2	2				
Cse1l					72	0.07	2	64
Csnk2a1	383	1.4	10	19	115	0.37	3	6
Csnk2b	238	1.32	6	14	80	0.32	2	3
Csrp2bp	66	0.08	2	9				
Ctbp2	452	0.67	7	68	154	0.24	4	8
Ctcf	87	0.14	3	5				
Ctr9	108	0.06	2	2				

	H3K27ac							
	Experiment 1				Experiment 2			
Protein	Mascot	emPAI	Unique pept.[a]	Pept. count[b]	Mascot	emPAI	Unique pept.[a]	Pept. count[b]
Brpf1								
Brpf3								
Cad								
Cald1								
Cbx7								
Cdc5l								
Cdc73	84	0.13	2	2	62	0.06	1	2
Cdca7					64	0.18	1	2
Cdca8					62	0.25	2	3
Cdk7								
Cdk9	61	0.18	2	2				
Cep95					45	0.04	1	14
Chaf1b	45	0.45	1	1				
Chd1	239	0.16	8	14	641	0.32	14	47
Chd2								
Chd8								
Ckb								
Coil								
Cpsf6					131	0.13	2	14
Creb1								
Cse1l								
Csnk2a1	106	0.27	3	5	106	0.27	3	4
Csnk2b					135	0.52	3	5
Csrp2bp								
Ctbp2	229	0.44	5	6	72	0.16	2	4
Ctcf								
Ctr9	91	0.06	2	2				

	H3K4me1							
	Experiment 1				Experiment 2			
Protein	Mascot	emPAI	Unique pept.[a]	Pept. count[b]	Mascot	emPAI	Unique pept.[a]	Pept. count[b]
Brpf1								
Brpf3								
Cad					204	0.08	6	9
Cald1								
Cbx7					56	0.19	1	1
Cdc5l					58	0.08	2	2
Cdc73								
Cdca7					51	0.18	1	4
Cdca8	97	0.23	2	5	56	0.25	2	2
Cdk7								
Cdk9								
Cep95	68	0.07	2	8				
Chaf1b	109	1.04	2	2				
Chd1	212	0.11	6	11	323	0.16	7	28
Chd2	96	0.05	3	7				
Chd8								
Ckb								
Coil								
Cpsf6								
Creb1								
Cse1l								
Csnk2a1	117	0.25	3	5	58	0.17	2	3
Csnk2b					51	0.15	1	2
Csrp2bp								
Ctbp2	309	0.51	6	9	188	0.34	4	6
Ctcf					89	0.09	2	4
Ctr9								

	H3K9me3							
	Experiment 1				Experiment 2			
Protein	Mascot	emPAI	Unique pept.[a]	Pept. count[b]	Mascot	emPAI	Unique pept.[a]	Pept. count[b]
Brpf1								
Brpf3								
Cad								
Cald1								
Cbx7								
Cdc5l								
Cdc73								
Cdca7								
Cdca8	344	1.17	7	24	392	1.17	7	40
Cdk7								
Cdk9								
Cep95								
Chaf1b								
Chd1					66	0.04	2	2
Chd2								
Chd8								
Ckb								
Coil					62	0.06	1	3
Cpsf6								
Creb1								
Cse1l								
Csnk2a1								
Csnk2b								
Csrp2bp								
Ctbp2					60	0.08	1	2
Ctcf								
Ctr9								

	GFP							
	Experiment 1				Experiment 2			
Protein	Mascot	emPAI	Unique pept.[a]	Pept. count[b]	Mascot	emPAI	Unique pept.[a]	Pept. count[b]
Brpf1								
Brpf3								
Cad								
Cald1								
Cbx7								
Cdc5l								
Cdc73								
Cdca7								
Cdca8								
Cdk7								
Cdk9								
Cep95								
Chaf1b								
Chd1								
Chd2								
Chd8								
Ckb								
Coil								
Cpsf6								
Creb1								
Cse1l								
Csnk2a1								
Csnk2b								
Csrp2bp								
Ctbp2								
Ctcf								
Ctr9								

	H3K4me3								H3K27ac								H3K4me1								H3K9me3								GFP							
	Experiment 1				Experiment 2				Experiment 1				Experiment 2				Experiment 1				Experiment 2				Experiment 1				Experiment 2				Experiment 1				Experiment 2			
Protein	Mascot	emPAI	Unique pept.[a]	Pept. count[b]	Mascot	emPAI	Unique pept.[a]	Pept. count[b]	Mascot	emPAI	Unique pept.[a]	Pept. count[b]	Mascot	emPAI	Unique pept.[a]	Pept. count[b]	Mascot	emPAI	Unique pept.[a]	Pept. count[b]	Mascot	emPAI	Unique pept.[a]	Pept. count[b]	Mascot	emPAI	Unique pept.[a]	Pept. count[b]	Mascot	emPAI	Unique pept.[a]	Pept. count[b]	Mascot	emPAI	Unique pept.[a]	Pept. count[b]	Mascot	emPAI	Unique pept.[a]	Pept. count[b]
Cul4b	262	0.26	7	9	79	0.07	2	2	239	0.22	6	8	195	0.14	4	4	229	0.13	4	8	275	0.22	7	11																
Ddb1	349	0.22	8	10	198	0.15	5	5	339	0.29	9	9	317	0.19	6	7	414	0.31	10	15	339	0.26	8	14																
Ddx47																									81	0.15	2	2	103	0.15	2	2								
Ddx54																	68	0.07	2	2																				
Dido1	120	0.04	3	12																																				
Dis3													92	0.1	3	3					84	0.1	3	3																
Dmap1	100	0.14	2	3													69	0.07	1	1																				
Dnajc9					48	0.13	1	3					89	0.27	2	5					137	0.27	2	4																
Dpf2									189	0.36	4	4	57	0.08	1	6	127	0.15	2	2	66	0.08	1	2																
Dppa2	255	0.86	6	33	157	0.36	3	13					69	0.11	1	9	85	0.21	2	6	152	0.36	3	19																
Dppa4	231	0.92	7	17	75	0.24	2	22									59	0.23	2	2	134	0.54	3	21																
Dpy30	50	0.36	1	4																																				
Dut	117	0.64	3	8	58	0.18	1	4	49	0.18	1	2	48	0.18	1	3	53	0.17	2	6	69	0.18	1	5																
Emsy	56	0.03	1	1																																				
Ep300									147	0.06	4	4	135	0.04	3	8																								
Ep400													101	0.03	3	3																								
Erh	71	0.74	2	2																																				
Esrrb									45	0.08	1	1	48	0.08	1	1	139	0.23	3	6	112	0.16	2	4																
Ezh2	99	0.09	2	5	73	0.04	1	2	47	0.04	1	1	98	0.04	1	2	79	0.04	1	2	141	0.14	3	4																
Fam60a	75	0.48	2	2																																				
Fbxl19					75	0.1	2	4																																
Fkbp4																	62	0.14	2	2																				
Gltscr2																	153	0.13	2	2																				
Glyr1	136	0.19	3	6	147	0.19	2	9	181	0.34	4	6	111	0.13	2	4	292	0.32	5	16	172	0.19	3	11	97	0.13	2	2												
Gm53					59	0.37	2	3																																
Gtf2e1																	76	0.15	2	4																				
Gtf2i									70	0.07	2	3					68	0.06	2	4	102	0.07	3	3																

	H3K4me3								H3K27ac								H3K4me1								H3K9me3								GFP							
	Experiment 1				Experiment 2				Experiment 1				Experiment 2				Experiment 1				Experiment 2				Experiment 1				Experiment 2				Experiment 1				Experiment 2			
Protein	Mascot	emPAI	Unique pept. [a]	Pept. count [b]	Mascot	emPAI	Unique pept. [a]	Pept. count [b]	Mascot	emPAI	Unique pept. [a]	Pept. count [b]	Mascot	emPAI	Unique pept. [a]	Pept. count [b]	Mascot	emPAI	Unique pept. [a]	Pept. count [b]	Mascot	emPAI	Unique pept. [a]	Pept. count [b]	Mascot	emPAI	Unique pept. [a]	Pept. count [b]	Mascot	emPAI	Unique pept. [a]	Pept. count [b]	Mascot	emPAI	Unique pept. [a]	Pept. count [b]	Mascot	emPAI	Unique pept. [a]	Pept. count [b]
Gtf3c1	55	0.02	1	1									53	0.02	1	1					97	0.03	2	4																
Hat1	88	0.15	2	4	45	0.07	1	2					62	0.07	1	2					63	0.07	1	6																
Hcfc1	137	0.09	4	7																																				
Hdac1	453	1.65	9	75	239	0.38	5	24	118	0.3	3	7	170	0.3	4	9	176	0.28	4	7	244	0.48	6	23	87	0.14	2	7	60	0.07	1	3								
Hdac2	222	0.79	5	52													114	0.2	3	6																				
Hdgfrp2													67	0.1	2	2																								
Hmga2					82	0.28	1	7					56	0.28	1	1									52	0.28	1	6	87	0.28	1	7								
Hmgb3	52	0.21	1	6													63	0.2	1	6																				
Hmgxb4	60	0.39	2	2																																				
Hp1bp3	45	0.12	1	3	104	0.39	3	6									142	0.51	4	7	116	0.24	2	9	132	0.19	4	8	172	0.27	4	11								
Incenp					152	0.11	3	9																	270	0.28	7	14	317	0.24	6	20								
Ing1	52	0.12	1	1																																				
Ing2	57	0.11	1	15	57	0.11	1	6																																
Ing3					70	0.08	1	1					74	0.08	1	1																								
Ing4	60	0.28	2	2																	48	0.13	1	1																
Ing5	168	0.87	4	22	71	0.29	1	2	46	0.13	1	3	63	0.13	1	1	72	0.27	2	3	73	0.13	1	2																
Ino80	147	0.06	3	5	79	0.04	3	3	140	0.09	4	5																												
Ints1																	69	0.03	2	17																				
Irgc																	68	0.08	2	12																				
Jarid2	283	0.17	8	13	309	0.14	5	11	102	0.05	2	2	128	0.05	2	4	281	0.19	7	11	424	0.23	8	19	70	0.03	2	2	104	0.05	2	3								
Kdm1a	94	0.08	2	2	91	0.04	1	1					57	0.04	1	1					86	0.04	1	1					53	0.04	1	1								
Kdm2a	435	0.24	9	43	158	0.08	3	15					66	0.06	2	2	51	0.03	1	2	62	0.03	1	3																
Kdm2b	267	0.16	6	13	71	0.02	2	5													64	0.02	1	3																
Kdm4a	67	0.06	2	3																																				
Kdm4c	861	0.87	18	86	647	0.43	11	48					119	0.06	2	4	73	0.06	2	2	270	0.2	6	16																
Kdm5a	61	0.04	2	2																																				
Kdm5b	519	0.3	13	38	424	0.2	9	16					107	0.04	2	3	68	0.05	1	5	347	0.18	8	18																

Protein	H3K4me3								H3K27ac								H3K4me1								H3K9me3								GFP							
	Experiment 1				Experiment 2				Experiment 1				Experiment 2				Experiment 1				Experiment 2				Experiment 1				Experiment 2				Experiment 1				Experiment 2			
	Mascot	emPAI	Unique pept. [a]	Pept. count [b]	Mascot	emPAI	Unique pept. [a]	Pept. count [b]	Mascot	emPAI	Unique pept. [a]	Pept. count [b]	Mascot	emPAI	Unique pept. [a]	Pept. count [b]	Mascot	emPAI	Unique pept. [a]	Pept. count [b]	Mascot	emPAI	Unique pept. [a]	Pept. count [b]	Mascot	emPAI	Unique pept. [a]	Pept. count [b]	Mascot	emPAI	Unique pept. [a]	Pept. count [b]	Mascot	emPAI	Unique pept. [a]	Pept. count [b]	Mascot	emPAI	Unique pept. [a]	Pept. count [b]
Kif2c																	70	0.09	2	2																				
Kif4																	103	0.05	2	2																				
Kifc5b													71	0.14	2	2	126	0.16	4	5	108	0.14	2	3																
Klf16	100	0.31	2	8																																				
Klf5									53	0.07	1	1	63	0.07	1	1					72	0.07	1	1																
L3mbtl2	94	0.15	3	3													111	0.14	3	3																				
Las1l																	106	0.12	3	3																				
Lasp1					85	0.58	2	5													57	0.26	1	2																
Lig1	147	0.15	4	4													55	0.03	1	1																				
Lig3													67	0.03	1	1					115	0.1	3	3																
Llph																	53	0.25	1	1																				
Lmna	96	0.1	2	3	155	0.16	3	3	130	0.16	3	4					155	0.15	3	5					451	0.55	9	12	290	0.41	6	8								
Lmnb1	424	0.72	10	29	807	1.25	12	58	805	1.95	19	39	794	1.25	13	37	1278	2.93	23	61	644	0.72	10	29	1483	4.34	28	88	1025	1.95	15	78	268	0.38	6	15	347	0.36	6	7
Lmnb2					173	0.24	4	12	170	0.24	4	6	154	0.24	3	8	297	0.42	6	13	173	0.17	4	6	701	1.24	15	22	435	0.71	9	30								
Lrwd1					220	0.28	5	13									59	0.05	1	2	93	0.16	3	3	163	0.28	5	9	224	0.42	6	21								
Lsm2													63	0.36	1	1																								
Luc7l3																	55	0.07	1	1																				
Max	56	0.21	1	9									45	0.21	1	1	72	0.2	1	1																				
Mbtd1	108	0.11	2	3																																				
Mdc1													143	0.06	3	10	100	0.06	3	3	96	0.04	2	6																
Meaf6	130	0.63	3	6	117	0.38	2	4	60	0.38	2	2									104	0.38	2	3																
Men1	333	0.45	7	24	106	0.18	3	8					50	0.06	1	2	55	0.05	1	2	232	0.25	4	16																
Mll2	575	0.23	16	35	518	0.18	10	21													146	0.04	3	8									61	0.01	2	4				
Morc2a									107	0.1	4	4																												
Morc3	865	0.91	21	64	626	0.48	12	35	225	0.18	6	8	437	0.3	8	22	141	0.1	3	4	501	0.38	10	24					59	0.03	1	2								
Mta3	141	0.28	4	6	173	0.2	3	4	171	0.28	5	5					189	0.26	4	4	164	0.28	4	4																
Mtf2	74	0.12	2	3	111	0.19	3	6	52	0.06	1	1	45	0.06	1	1	123	0.18	3	3	161	0.26	4	6					62	0.12	2	2								

	H3K4me3								H3K27ac								H3K4me1								H3K9me3								GFP							
	Experiment 1				Experiment 2				Experiment 1				Experiment 2				Experiment 1				Experiment 2				Experiment 1				Experiment 2				Experiment 1				Experiment 2			
Protein	Mascot	emPAI	Unique pept.[a]	Pept. count[b]	Mascot	emPAI	Unique pept.[a]	Pept. count[b]	Mascot	emPAI	Unique pept.[a]	Pept. count[b]	Mascot	emPAI	Unique pept.[a]	Pept. count[b]	Mascot	emPAI	Unique pept.[a]	Pept. count[b]	Mascot	emPAI	Unique pept.[a]	Pept. count[b]	Mascot	emPAI	Unique pept.[a]	Pept. count[b]	Mascot	emPAI	Unique pept.[a]	Pept. count[b]	Mascot	emPAI	Unique pept.[a]	Pept. count[b]	Mascot	emPAI	Unique pept.[a]	Pept. count[b]
Mycn	66	0.16	2	3					66	0.16	2	3																												
Myst2	327	0.75	9	30	268	0.36	5	10	201	0.36	6	7	296	0.36	5	11	170	0.27	4	10	316	0.5	7	13					89	0.11	2	2								
Myst3	293	0.1	6	11	167	0.05	3	4																																
Myst4	70	0.04	2	2	81	0.04	2	3																																
Nacc1					47	0.07	1	2					111	0.13	2	2	130	0.19	3	5	52	0.07	1	1																
Nasp													58	0.04	1	1					46	0.04	1	1																
Nfrkb									58	0.05	2	2																												
Nolc1																	57	0.1	2	4																				
Nrf1	66	0.15	2	2																																				
Nsd1	62	0.02	2	3					138	0.05	4	4	96	0.02	2	3	190	0.05	4	9	163	0.04	3	11																
Nudt13																	62	0.19	2	3																				
Nudt21	123	0.96	4	6																																				
Numa1	402	0.13	9	25	339	0.3	6	17	431	0.18	11	17	457	0.11	7	17	685	0.19	13	31	666	0.39	11	27	284	0.1	6	10	105	0.09	2	5								
Oct4	94	0.32	3	6					66	0.2	2	2					170	0.42	4	7					51	0.1	1	2												
Ogt	208	0.2	6	6	183	0.1	3	3					46	0.03	1	1					178	0.1	3	3																
Pak1ip1																	134	0.37	4	4																				
Parp2																	86	0.05	2	4																				
Parp9																													58	0.08	2	2								
Patz1	107	0.1	3	10													74	0.09	2	3																				
Pbrm1	66	0.04	2	3					220	0.11	6	8	174	0.11	5	9	146	0.08	4	6	218	0.14	6	11																
Pds5a													99	0.05	2	5	162	0.09	4	6																				
Pds5b					78	0.04	2	2					56	0.02	1	1					83	0.04	2	2																
Phc1																					80	0.07	2	2																
Phf16					69	0.08	2	5																																
Phf20																					60	0.03	1	1																
Phf23	148	0.4	2	19	196	0.66	3	24													56	0.18	1	6																
Phf8	345	0.33	7	24	144	0.13	3	8	51	0.04	1	1																												

Protein	H3K4me3								H3K27ac							
	Experiment 1				Experiment 2				Experiment 1				Experiment 2			
	Mascot	emPAI	Unique pept. [a]	Pept. count [b]	Mascot	emPAI	Unique pept. [a]	Pept. count [b]	Mascot	emPAI	Unique pept. [a]	Pept. count [b]	Mascot	emPAI	Unique pept. [a]	Pept. count [b]
Phip	152	0.05	4	7	63	0.02	1	1	134	0.07	4	4	187	0.07	4	6
Pin1													57	0.21	1	3
Pnp	129	0.39	3	5												
Polr1c	174	0.6	5	8												
Polr2a	460	0.18	11	21	321	0.11	6	9	333	0.12	8	9	498	0.14	9	15
Polr2b	709	0.67	18	54	258	0.18	6	14	407	0.34	11	17	421	0.31	10	17
Polr2c	308	0.93	7	16	147	0.21	2	2					158	0.32	3	5
Polr2e	252	1.37	6	12	100	0.33	2	2	57	0.33	2	2	71	0.15	1	2
Polr2g	114	0.44	2	2												
Ppp2cb																
Prdm10	63	0.06	2	2									87	0.06	2	5
Prdm2																
Prrc2c																
Psma5	79	0.14	1	1												
Ptcd3																
Qser1	135	0.06	4	4	46	0.02	1	1								
Rad21													57	0.05	1	1
Rars																
Rbbp4	697	2.02	14	108	476	1	9	71	288	0.62	7	13	304	0.62	6	28
Rbbp5	144	0.27	4	9												
Rbl1																
Rbpj	169	0.3	4	8	142	0.3	4	9								
Rfc3					71	0.09	1	1					74	0.19	2	3
Rfc4	94	0.3	3	4	52	0.09	1	1	103	0.3	3	3				
Rnf2	212	0.6	5	12	52	0.1	1	1								
Rsf1	81	0.07	2	3	76	0.02	1	4	192	0.09	4	7	279	0.12	5	16
Ruvbl2	729	2.3	14	92	240	0.42	5	7	497	1.02	9	28	716	2.8	15	24

Protein	H3K4me1								H3K9me3							
	Experiment 1				Experiment 2				Experiment 1				Experiment 2			
	Mascot	emPAI	Unique pept. [a]	Pept. count [b]	Mascot	emPAI	Unique pept. [a]	Pept. count [b]	Mascot	emPAI	Unique pept. [a]	Pept. count [b]	Mascot	emPAI	Unique pept. [a]	Pept. count [b]
Phip	248	0.1	7	9	295	0.15	7	17								
Pin1					48	0.21	1	2								
Pnp																
Polr1c																
Polr2a	129	0.05	3	3	159	0.07	4	5								
Polr2b	63	0.05	2	2	148	0.08	3	4								
Polr2c					100	0.21	2	2								
Polr2e					55	0.15	2	2								
Polr2g																
Ppp2cb					77	0.22	2	2								
Prdm10																
Prdm2					66	0.04	2	4								
Prrc2c	65	0.02	2	2												
Psma5	45	0.13	1	1												
Ptcd3	235	0.24	5	7												
Qser1																
Rad21	51	0.05	1	1												
Rars	89	0.09	2	2												
Rbbp4	507	1.18	12	25	488	1	9	48	186	0.41	5	8	191	0.32	4	19
Rbbp5																
Rbl1	62	0.06	2	3												
Rbpj					222	0.39	5	14	64	0.14	2	2	61	0.14	2	2
Rfc3																
Rfc4	47	0.08	1	1	137	0.3	4	4								
Rnf2					176	0.6	4	18								
Rsf1	127	0.09	3	9	325	0.14	6	19								
Ruvbl2	291	0.49	6	10	170	0.32	4	4	130	0.32	4	4				

Protein	GFP							
	Experiment 1				Experiment 2			
	Mascot	emPAI	Unique pept. [a]	Pept. count [b]	Mascot	emPAI	Unique pept. [a]	Pept. count [b]
Phip								
Pin1								
Pnp								
Polr1c								
Polr2a								
Polr2b								
Polr2c								
Polr2e								
Polr2g								
Ppp2cb								
Prdm10								
Prdm2								
Prrc2c								
Psma5								
Ptcd3								
Qser1								
Rad21								
Rars								
Rbbp4	147	0.23	4	5				
Rbbp5								
Rbl1								
Rbpj								
Rfc3								
Rfc4								
Rnf2								
Rsf1								
Ruvbl2								

	H3K4me3								H3K27ac								H3K4me1								H3K9me3								GFP							
	Experiment 1				Experiment 2				Experiment 1				Experiment 2				Experiment 1				Experiment 2				Experiment 1				Experiment 2				Experiment 1				Experiment 2			
Protein	Mascot	emPAI	Unique pept.[a]	Pept. count[b]	Mascot	emPAI	Unique pept.[a]	Pept. count[b]	Mascot	emPAI	Unique pept.[a]	Pept. count[b]	Mascot	emPAI	Unique pept.[a]	Pept. count[b]	Mascot	emPAI	Unique pept.[a]	Pept. count[b]	Mascot	emPAI	Unique pept.[a]	Pept. count[b]	Mascot	emPAI	Unique pept.[a]	Pept. count[b]	Mascot	emPAI	Unique pept.[a]	Pept. count[b]	Mascot	emPAI	Unique pept.[a]	Pept. count[b]	Mascot	emPAI	Unique pept.[a]	Pept. count[b]
Samd1	206	0.3	4	22	139	0.22	3	9																																
Sap130	215	0.22	5	14	183	0.14	4	5																																
Sap30	136	0.57	3	9																																				
Sap30l	53	0.18	1	2																																				
Sbno1																	59	0.02	2	3																				
Sephs1	83	0.28	2	3									47	0.09	1	1																								
Sf1	62	0.27	2	2																																				
Sf3b1																	150	0.07	3	4																				
Shprh	84	0.04	2	2	75	0.04	2	2					46	0.02	1	2					118	0.06	3	6																
Sin3a	1717	1.85	36	143	1192	0.82	22	64	178	0.13	4	6	278	0.16	5	9	321	0.21	8	8	695	0.38	12	19																
Sin3b	115	0.09	3	4	118	0.09	3	4																																
Skp1a	110	0.45	2	11	71	0.45	2	5					61	0.45	2	3					61	0.2	1	2																
Smarca4	430	0.22	9	15	106	0.06	3	5	530	0.25	11	18	742	0.3	12	33	510	0.16	8	13	580	0.27	11	29	56	0.02	1	1	57	0.04	2	2								
Smarcb1									72	0.18	2	2									63	0.09	1	3																
Smarcc1	131	0.09	4	5	137	0.11	3	4	495	0.47	12	15	535	0.45	10	20	317	0.25	9	10	471	0.36	8	15	51	0.03	1	1	98	0.11	3	3								
Smarcd1									280	0.54	7	7	240	0.45	6	8	152	0.34	4	5	197	0.36	5	5																
Smc1a	126	0.11	4	4	56	0.05	2	2	240	0.19	7	7	66	0.05	2	2	452	0.3	11	12	180	0.13	4	6	141	0.11	4	4												
Smc6																	82	0.05	2	2																				
Smchd1									78	0.03	2	2					137	0.05	3	5					119	0.05	3	4	51	0.02	1	2								
Spin1	248	1.31	6	45	62	0.13	1	17	46	0.13	1	4	77	0.13	1	8	48	0.13	1	4	58	0.13	1	9																
Srrt	61	0.07	2	2					91	0.07	3	3					130	0.11	3	3	83	0.04	1	1																
Ssrp1	582	0.93	12	66	474	0.42	7	54	585	0.93	13	39	687	0.93	11	65	685	0.88	13	41	511	0.57	8	88	219	0.22	4	14	296	0.35	6	25	75	0.11	2	3	150	0.15	3	7
Suds3	234	0.6	6	8	105	0.21	2	2																																
Supt4h2	77	0.67	2	4																																				
Supt5h	386	0.31	10	13	265	0.13	4	9	191	0.16	5	5	281	0.16	5	13	72	0.06	2	2	155	0.06	2	4																
Supt6h	207	0.12	6	7	90	0.02	1	1	177	0.1	5	5	249	0.08	4	4																								
Suv39h2																									118	0.22	4	7	167	0.39	4	7								

	H3K4me3								H3K27ac								H3K4me1								H3K9me3								GFP							
	Experiment 1				Experiment 2				Experiment 1				Experiment 2				Experiment 1				Experiment 2				Experiment 1				Experiment 2				Experiment 1				Experiment 2			
Protein	Mascot	emPAI	Unique pept. [a]	Pept. count [b]	Mascot	emPAI	Unique pept. [a]	Pept. count [b]	Mascot	emPAI	Unique pept. [a]	Pept. count [b]	Mascot	emPAI	Unique pept. [a]	Pept. count [b]	Mascot	emPAI	Unique pept. [a]	Pept. count [b]	Mascot	emPAI	Unique pept. [a]	Pept. count [b]	Mascot	emPAI	Unique pept. [a]	Pept. count [b]	Mascot	emPAI	Unique pept. [a]	Pept. count [b]	Mascot	emPAI	Unique pept. [a]	Pept. count [b]	Mascot	emPAI	Unique pept. [a]	Pept. count [b]
Suz12	229	0.37	6	11	251	0.25	5	11	73	0.09	2	2	193	0.14	4	6	294	0.29	6	10	455	0.5	8	19					144	0.14	3	6								
Taf1	100	0.05	3	3																																				
Taf2	149	0.12	4	5					47	0.03	1	1																												
Taf3	79	0.04	1	7					68	0.04	1	1																												
Taf4a	144	0.22	4	4																																				
Taf5	65	0.09	2	2																																				
Taf6	230	0.29	5	6	136	0.11	2	3	97	0.11	3	3	168	0.17	3	3																								
Taf7	84	0.2	2	5	70	0.11	1	1	48	0.11	1	1	59	0.11	1	2																								
Taldo1													50	0.1	1	1					55	0.21	2	2																
Tbrg4																	67	0.17	2	3																				
Tcea1	78	0.23	2	6					133	0.52	4	4					47	0.1	1	2																				
Tcerg1					57	0.03	1	1					125	0.06	2	3					63	0.03	1	2																
Tcof1																									75	0.03	1	1												
Tead1													56	0.08	1	2					50	0.08	1	1																
Tlk2																	67	0.08	2	3	47	0.04	1	1																
Trim24									265	0.24	7	7	343	0.25	7	8					90	0.07	2	2																
Trim33									123	0.09	3	3	131	0.06	2	3																								
Trp53													52	0.11	1	1																								
Trrap	204	0.06	5	5	148	0.04	4	4	98	0.04	3	3	314	0.05	6	9					52	0.01	1	1																
Uba1													64	0.06	2	2					83	0.06	2	2																
Ube2h																	56	0.47	2	3																				
Ubtf	732	1.07	19	73	457	0.5	9	34	374	0.5	10	14	359	0.38	8	18	342	0.35	8	14	457	0.5	10	28																
Usp48	195	0.13	4	11	162	0.16	3	7					143	0.16	3	7	457	0.29	10	19	223	0.13	4	18	52	0.05	1	1	53	0.05	1	2								
Utp14b																	110	0.13	3	5																				
Vmn2r100					59	0.04	2	3													63	0.12	2	3																
Wdr18	52	0.08	1	1													119	0.24	3	3					68	0.08	2	2	49	0.08	1	2								
Wdr5	202	0.57	4	22	170	0.62	5	12	86	0.21	2	3	133	0.33	3	10					115	0.33	3	4																

	H3K4me3								H3K27ac								H3K4me1								H3K9me3								GFP							
	Experiment 1				Experiment 2				Experiment 1				Experiment 2				Experiment 1				Experiment 2				Experiment 1				Experiment 2				Experiment 1				Experiment 2			
Protein	Mascot	emPAI	Unique pept. [a]	Pept. count [b]	Mascot	emPAI	Unique pept. [a]	Pept. count [b]	Mascot	emPAI	Unique pept. [a]	Pept. count [b]	Mascot	emPAI	Unique pept. [a]	Pept. count [b]	Mascot	emPAI	Unique pept. [a]	Pept. count [b]	Mascot	emPAI	Unique pept. [a]	Pept. count [b]	Mascot	emPAI	Unique pept. [a]	Pept. count [b]	Mascot	emPAI	Unique pept. [a]	Pept. count [b]	Mascot	emPAI	Unique pept. [a]	Pept. count [b]	Mascot	emPAI	Unique pept. [a]	Pept. count [b]
Wdr55					57	0.09	2	2																																
Zfp280c					116	0.09	4	5																	161	0.24	4	11	190	0.19	4	10								
Zfp281	74	0.08	2	2																																				
Zfp462																	53	0.01	1	1					68	0.03	2	2												
Zic5	64	0.06	1	1																																				
Zmynd8									57	0.03	2	2	101	0.06	2	6					55	0.07	1	7																
Znf512					153	0.25	4	10									160	0.3	5	7	91	0.18	3	3	191	0.32	5	13	161	0.18	3	14								
Zscan10	60	0.08	2	2																	93	0.17	2	2																

[a] Number of unique, non-redundant peptides identified for the specified protein

[b] Number of peptide spectra counted for the specified protein

Engelen, Brandsma, Supplementary Table 2

Supplementary Table 3. ChIP-MS identified complex subunits and predictions

Complex / subunits	H3K4me3 avrg emPAI	H3K27ac avrg emPAI	H3K4me1 avrg emPAI	H3K9me3 avrg emPAI	GFP avrg emPAI	H3K27ac ratio	Prediction[a]
BAF complex	0,04	0,25	0,17	0,01		1,43	Enhancer
Arid1a (Baf250a)		0,07	0,06				Enhancer
Dpf2 (Baf45d)		0,22	0,12				Enhancer
Pbrm1 (Baf180)	0,02	0,11	0,11				Enhancer
Smarca4 (Brg1)	0,14	0,28	0,22	0,03			Enhancer
Smarcb1 (Baf47)		0,09	0,05				Enhancer
Smarcc1 (Baf155)	0,10	0,46	0,31	0,07			Enhancer
Smarcd1 (Baf60a)		0,50	0,35				Enhancer
Sin3 complex	0,32	0,01	0,03			0,04	Promoter
Sin3a	1,34	0,15	0,30				Promoter
Sin3b	0,09						Promoter
Arid4a (Rbbp1)	0,12						Promoter
Brms1	0,14						Promoter
Brms1l	0,47						Promoter
Fam60a	0,24						Promoter
Ing1	0,06						Promoter
Ing2	0,11						Promoter
Sap130	0,18						Promoter
Sap30	0,29						Promoter
Sap30l	0,09						Promoter
Suds3	0,41						Promoter
PRC1 complex	0,15		0,14			0	Promoter
Cbx7	0,10		0,10				Promoter
Phc1			0,04				Enhancer
Rnf2	0,35		0,30				Promoter
PRC2 complex	0,17	0,07	0,23	0,04		0,29	Enhancer
Ezh2	0,07	0,04	0,09				Enhancer
Jarid2	0,16	0,05	0,21	0,04			Enhancer
Mtf2	0,16	0,06	0,22	0,06			Enhancer
Suz12	0,31	0,12	0,40	0,07			Enhancer
TFIID complex	0,09	0,04				0,47	Promoter
Taf1	0,03						Promoter
Taf2	0,06	0,02					Promoter
Taf3	0,02	0,02					Promoter
Taf4a	0,11						Promoter
Taf5	0,05						Promoter
Taf6	0,20	0,14					Promoter
Taf7	0,16	0,11					Promoter
Aurora Kinase complex	0,19	0,08	0,11	0,69			Heterochromatin
Aurkb	0,22	0,10	0,10	0,65			Heterochromatin
Cdca8	0,28	0,13	0,24	1,17			Heterochromatin
Incenp	0,06			0,26			Heterochromatin

Complex / subunits	H3K4me3 avrg emPAI	H3K27ac avrg emPAI	H3K4me1 avrg emPAI	H3K9me3 avrg emPAI	GFP avrg emPAI	H3K27ac ratio	Prediction[a]
Pol II complex	0,44	0,17	0,06			0,39	Promoter
Polr2a	0,15	0,13	0,06				Promoter
Polr2b	0,43	0,33	0,07				Promoter
Polr2c	0,57	0,16	0,11				Promoter
Polr2e	0,85	0,24	0,08				Promoter
Polr2g	0,22						Promoter
MLL complex	0,34	0,04	0,05			0,13	Promoter
Mll2	0,21		0,02		0,01		Promoter
Ash2l	0,25						Promoter
Bap18	0,63						Promoter
Dpy30	0,18						Promoter
Hcfc1	0,05						Promoter
Men1	0,32	0,03	0,15				Promoter
Rbbp5	0,14						Promoter
Wdr5	0,60	0,27	0,17				Promoter
Trrap/Ep400 complex	0,30	0,39	0,09	0,03	0,00	1,33	Promoter
Trrap	0,05	0,05	0,01				Promoter
Ep400		0,02					Unclear
Dmap1	0,07		0,04				Promoter
Ruvbl2	1,36	1,91	0,41	0,16			Promoter
HBO1/MOZ/MORF complex	0,20	0,07	0,09	0,01		0,34	Promoter
Myst2	0,56	0,36	0,39	0,06			Promoter
Myst3	0,08						Promoter
Myst4	0,04						Promoter
Brd1	0,03	0,05	0,10				Enhancer
Brpf1	0,16						Promoter
Brpf3	0,03						Promoter
Meaf6	0,51	0,19	0,19				Promoter
Ing4	0,14		0,07				Promoter
Ing5	0,58	0,13	0,20				Promoter
Phf16	0,04						Promoter
Cohesin complex	0,04	0,07	0,12	0,03		0,60	Enhancer
Rad21		0,03	0,03				Enhancer
Smc1a	0,08	0,12	0,22	0,06			Enhancer
DSIF complex	0,28	0,08	0,03			0,29	Promoter
Supt4h2	0,34						Promoter
Supt5h	0,22	0,16	0,06				Promoter

[a] Prediction of genome localization based on our ChIP-MS criteria

Supplementary Table 4. Dppa2 target genes

Gene symbol	Ensembl gene ID	Tissue/cells with highest expression	FC over Embryonic stem cells
Pdgfb	ENSMUSG00000000489	Macrophage bone marrow 2hr LPS	83.7
Gnmt	ENSMUSG00000002769	Liver	3404.7
Gstm5	ENSMUSG00000004032	Testis	247.1
Taf7l	ENSMUSG00000009596	Placenta	633.1
Slc47a1	ENSMUSG00000010122	Kidney	64.5
Dazl	ENSMUSG00000010592	Testis	27.1
Mov10l1	ENSMUSG00000015365	Testis	26.4
Cd83	ENSMUSG00000015396	Macrophage peri LPS thio 1hrs	2432.4
Nkx2-5	ENSMUSG00000015579	Heart	26.8
Nuak1	ENSMUSG00000020032	MEF	46.2
Sycp3	ENSMUSG00000020059	Testis	19.2
Fgf22	ENSMUSG00000020327	Epidermis	1.8
Nipal4	ENSMUSG00000020411	Epidermis	56.4
Myocd	ENSMUSG00000020542	Umbilical cord	121.9
Cmpk2	ENSMUSG00000020638	Macrophage peri LPS thio 7hrs	2480.9
Rasgrf2	ENSMUSG00000021708	Hypothalamus	160.9
Ddx4	ENSMUSG00000021758	Testis	13.1
Galnt14	ENSMUSG00000024064	Kidney	11.1
Lipo1	ENSMUSG00000024766	Lacrimal gland	33.4
Rin1	ENSMUSG00000024883	Nucleus accumbens	19.1
Tdrd1	ENSMUSG00000025081	Testis	82.6
Syce1	ENSMUSG00000025480	Testis	24.4
1500015O10Rik	ENSMUSG00000026051	Osteoblast day21	2756
Tnfrsf11a	ENSMUSG00000026321	RAW 264 7	79.7
Cybrd1	ENSMUSG00000027015	Stomach	58.2
Bfsp1	ENSMUSG00000027420	Lens	5785.3
Adad1	ENSMUSG00000027719	Testis	1618.9
Sycp1	ENSMUSG00000027855	Testis	108.1
Hormad1	ENSMUSG00000028109	Not Available	Not available
Spaca1	ENSMUSG00000028264	Testis	1382.4
Slc10a4	ENSMUSG00000029219	Not Available	Not available
Figla	ENSMUSG00000030001	Lens	1.7
Mesp2	ENSMUSG00000030543	Testis	3.3
Chst15	ENSMUSG00000030930	Mast cells IgE	73.9
Cryab	ENSMUSG00000032060	Lens	157.1
Itga11	ENSMUSG00000032243	Not Available	Not available
Kank1	ENSMUSG00000032702	Epidermis	61.2
Ptprm	ENSMUSG00000033278	Lung	118.1
Cdcp1	ENSMUSG00000035498	Cornea	36.9
C530008M17Rik	ENSMUSG00000036377	Testis	50.3
Kcna6	ENSMUSG00000038077	Cerebral cortex prefrontal	26.2
Spon1	ENSMUSG00000038156	Lens	391.8
Mpp6	ENSMUSG00000038388	Mast cells IgE	3.6
Tspyl5	ENSMUSG00000038984	Nucleus accumbens	98

Gene symbol	Ensembl gene ID	Tissue/cells with highest expression	FC over Embryonic stem cells
D1Pas1	ENSMUSG00000039224	Testis	6.5
Dusp26	ENSMUSG00000039661	Dorsal root ganglia	95.3
Fkbp6	ENSMUSG00000040013	Testis	12.6
Sec1	ENSMUSG00000040364	Testis	21.3
Mael	ENSMUSG00000040629	Testis	89.3
Rsph6a	ENSMUSG00000040866	Testis	262.4
Prima1	ENSMUSG00000041669	Neuro2a	9.9
Uggt2	ENSMUSG00000042104	Testis	9.2
Abcb4	ENSMUSG00000042476	Liver	190.9
Disc1	ENSMUSG00000043051	Not Available	Not available
Npy5r	ENSMUSG00000044014	Nucleus accumbens	11
Pcsk9	ENSMUSG00000044254	mIMCD.3	20.9
Mettl24	ENSMUSG00000045555	Lung	11.5
Olig3	ENSMUSG00000045591	Thymocyte DP CD4.CD8.	2
She	ENSMUSG00000046280	Lung	19.1
Gm11554	ENSMUSG00000048294	Embryonic stem cells Bruce4+ V26, average	1
Zfp697	ENSMUSG00000050064	Kidney	40.5
Tacstd2	ENSMUSG00000051397	Epidermis	640.2
Kcnf1	ENSMUSG00000051726	Olfactory bulb	263.7
Fes	ENSMUSG00000053158	Mast cells	99.2
Sh2d4a	ENSMUSG00000053886	Stomach	106.8
Kbtbd13	ENSMUSG00000054978	Skeletal muscle	29.6
H2-Q5	ENSMUSG00000055413	T.cells CD8.	783.5
Gulp1	ENSMUSG00000056870	Retinal pigment epithelium	87.5
Unc13d	ENSMUSG00000057948	Mast cells	73.8
Kl	ENSMUSG00000058488	Kidney	1051.7
Kcng2	ENSMUSG00000059852	Heart	80.1
Nkpd1	ENSMUSG00000060621	Stomach	8.2
Rnf217	ENSMUSG00000063760	C2C12	25.6
Zar1	ENSMUSG00000063935	Ovary	4.1
Dmrtc1c2	ENSMUSG00000067561	Not Available	Not available
4930444P10Rik	ENSMUSG00000067795	Testis	249.5
Slc25a31	ENSMUSG00000069041	Testis	27.1
Atxn1l	ENSMUSG00000069895	Ciliary bodies	3.9
Hsf5	ENSMUSG00000070345	Testis	303.5
Zfp783	ENSMUSG00000072653	Retinal pigment epithelium	22.4
Klhl40	ENSMUSG00000074001	Skeletal muscle	253
Tspyl3	ENSMUSG00000074671	T.cells CD8.	38.2
Chst14	ENSMUSG00000074916	X3T3.L1	9.7
Vgll3	ENSMUSG00000091243	Osteoblast day5	16.7

Supplementary Table 5. Accession numbers of used ChIP-seq data sets

**Histone modifications**	**GEO dataset accession number**
H3K27ac	GSE24165
H3K4me1	GSE24165
H3K4me3	GSE24165
H3K9me3	GSE12241
Input	GSE24165

**Protein**	**GEO dataset accession number**
Atrx	GSE22162
Brd4	GSE36561
Cbx7	GSE42466
Ctcf	GSE49847
Ctr9	GSE20530
Esrrb	GSE11431
Ezh2	GSE49178
GFP	GSE11431
Hdac1	GSE27844
Hdac2	GSE27844
Jarid2	GSE19708
Kdm1a	GSE27844
Kdm2a	GSE21202
Kdm5b	GSE31968
Mtf2	GSE16526
Mycn	GSE11431
Nanog	GSE44286
Oct4	GSE44286
P300	GSE49847
Polr2a	GSE49847
Rad21	GSE33346
Rbbp5	GSE22934
Rnf2	GSE26680
Smarca4	GSE14344
Smc1a	GSE22557
Supt5h	GSE20485
Suz12	GSE48122
Taf1	GSE31270
Taf3	GSE30959
Wdr5	GSE22934

**Protein**	**Bioproject accession number**
Tcea1	PRJEB2674

References for pluripotency phenotype of factors identified by ChIP-MS

Arid1a[1], Ash2l[2], Aurkb[3], Banf1[4], Bptf1[5], Cbx7[6], Cdc73[7], Cdk9[8], Chaf1b[9], Chd1[10], Ctbp2[11], Ctr9[8,12], Dmap1[13], Dppa2[14], Dppa4[14], Dpy30[15], Ep300[16], Ep400[13], Esrrb[17], Ezh2[18], Hcfc1[19], Hdac1[20], Ing5[8], Ino80[19], Jarid2[21], Kdm1a[22], Kdm2b[23], Kdm4c[24], Kdm5b[25], Klf5[26], L3mbtl2[27], Max[28], Mtf2[29], Mycn[30], Myst2[31], Myst3[31], Nacc1[32], Nfrkb[19], Oct4[33], Ogt[34], Phf20[35], Phf23[12], Pin1[36], , Rad21[37], Rbbp5[15], Rnf2[8,13,37], Ruvbl2[13], Sin3a[38], Smarca4[39], Smarcb1[40], Smarcc1[13], Smc1a[8,13], Smc6[12,13], Supt4h2[13], Suv39h2[19], Suz12[41], Taf1[42], Taf2[42], Taf4a[42], Taf5[42], Taf6[42], Taf3[42,43] , Taf7[19], Tpr[13,19], Trrap[13], Wdr5[44], Zfp281[32,45], Zscan10[46].

References

1. Gao, X. et al. ES cell pluripotency and germ-layer formation require the SWI/SNF chromatin remodeling component BAF250a. *Proc Natl Acad Sci U S A* **105**, 6656-61 (2008).
2. Wan, M. et al. The trithorax group protein Ash2l is essential for pluripotency and maintaining open chromatin in embryonic stem cells. *J Biol Chem* **288**, 5039-48 (2013).
3. Lee, D.F. et al. Regulation of embryonic and induced pluripotency by aurora kinase-p53 signaling. *Cell Stem Cell* **11**, 179-94 (2012).
4. Cox, J.L. et al. Banf1 is required to maintain the self-renewal of both mouse and human embryonic stem cells. *J Cell Sci* **124**, 2654-65 (2011).
5. Landry, J. et al. Essential role of chromatin remodeling protein Bptf in early mouse embryos and embryonic stem cells. *PLoS Genet* **4**, e1000241 (2008).
6. O'Loghlen, A. et al. MicroRNA regulation of Cbx7 mediates a switch of Polycomb orthologs during ESC differentiation. *Cell Stem Cell* **10**, 33-46 (2012).
7. Wang, P. et al. Parafibromin, a component of the human PAF complex, regulates growth factors and is required for embryonic development and survival in adult mice. *Mol Cell Biol* **28**, 2930-40 (2008).
8. Hu, G. et al. A genome-wide RNAi screen identifies a new transcriptional module required for self-renewal. *Genes Dev* **23**, 837-48 (2009).
9. Bilodeau, S., Kagey, M.H., Frampton, G.M., Rahl, P.B. & Young, R.A. SetDB1 contributes to repression of genes encoding developmental regulators and maintenance of ES cell state. *Genes Dev* **23**, 2484-9 (2009).
10. Gaspar-Maia, A. et al. Chd1 regulates open chromatin and pluripotency of embryonic stem cells. *Nature* **460**, 863-8 (2009).
11. Tarleton, H.P. & Lemischka, I.R. Delayed differentiation in embryonic stem cells and mesodermal progenitors in the absence of CtBP2. *Mech Dev* **127**, 107-19 (2010).
12. Ding, L. et al. A genome-scale RNAi screen for Oct4 modulators defines a role of the Paf1 complex for embryonic stem cell identity. *Cell Stem Cell* **4**, 403-15 (2009).
13. Fazzio, T.G., Huff, J.T. & Panning, B. An RNAi screen of chromatin proteins identifies Tip60-p400 as a regulator of embryonic stem cell identity. *Cell* **134**, 162-74 (2008).
14. Maldonado-Saldivia, J. et al. Dppa2 and Dppa4 are closely linked SAP motif genes restricted to pluripotent cells and the germ line. *Stem Cells* **25**, 19-28 (2007).
15. Jiang, H. et al. Role for Dpy-30 in ES cell-fate specification by regulation of H3K4 methylation within bivalent domains. *Cell* **144**, 513-25 (2011).
16. Zhong, X. & Jin, Y. Critical roles of coactivator p300 in mouse embryonic stem cell differentiation and Nanog expression. *J Biol Chem* **284**, 9168-75 (2009).

17. Ivanova, N. et al. Dissecting self-renewal in stem cells with RNA interference. *Nature* **442**, 533-8 (2006).
18. Shen, X. et al. EZH1 mediates methylation on histone H3 lysine 27 and complements EZH2 in maintaining stem cell identity and executing pluripotency. *Mol Cell* **32**, 491-502 (2008).
19. Chia, N.Y. et al. A genome-wide RNAi screen reveals determinants of human embryonic stem cell identity. *Nature* **468**, 316-20 (2010).
20. Dovey, O.M., Foster, C.T. & Cowley, S.M. Histone deacetylase 1 (HDAC1), but not HDAC2, controls embryonic stem cell differentiation. *Proc Natl Acad Sci U S A* **107**, 8242-7 (2010).
21. Shen, X. et al. Jumonji modulates polycomb activity and self-renewal versus differentiation of stem cells. *Cell* **139**, 1303-14 (2009).
22. Whyte, W.A. et al. Enhancer decommissioning by LSD1 during embryonic stem cell differentiation. *Nature* **482**, 221-5 (2012).
23. He, J. et al. Kdm2b maintains murine embryonic stem cell status by recruiting PRC1 complex to CpG islands of developmental genes. *Nat Cell Biol* **15**, 373-84 (2013).
24. Loh, Y.H., Zhang, W., Chen, X., George, J. & Ng, H.H. Jmjd1a and Jmjd2c histone H3 Lys 9 demethylases regulate self-renewal in embryonic stem cells. *Genes Dev* **21**, 2545-57 (2007).
25. Xie, L. et al. KDM5B regulates embryonic stem cell self-renewal and represses cryptic intragenic transcription. *EMBO J* **30**, 1473-84 (2011).
26. Ema, M. et al. Kruppel-like factor 5 is essential for blastocyst development and the normal self-renewal of mouse ESCs. *Cell Stem Cell* **3**, 555-67 (2008).
27. Qin, J. et al. The polycomb group protein L3mbtl2 assembles an atypical PRC1-family complex that is essential in pluripotent stem cells and early development. *Cell Stem Cell* **11**, 319-32 (2012).
28. Hishida, T. et al. Indefinite self-renewal of ESCs through Myc/Max transcriptional complex-independent mechanisms. *Cell Stem Cell* **9**, 37-49 (2011).
29. Walker, E. et al. Polycomb-like 2 associates with PRC2 and regulates transcriptional networks during mouse embryonic stem cell self-renewal and differentiation. *Cell Stem Cell* **6**, 153-66 (2010).
30. Chappell, J., Sun, Y., Singh, A. & Dalton, S. MYC/MAX control ERK signaling and pluripotency by regulation of dual-specificity phosphatases 2 and 7. *Genes Dev* **27**, 725-33 (2013).
31. Kagey, M.H. et al. Mediator and cohesin connect gene expression and chromatin architecture. *Nature* **467**, 430-5 (2010).
32. Wang, J. et al. A protein interaction network for pluripotency of embryonic stem cells. *Nature* **444**, 364-8 (2006).
33. Niwa, H., Miyazaki, J. & Smith, A.G. Quantitative expression of Oct-3/4 defines differentiation, dedifferentiation or self-renewal of ES cells. *Nat Genet* **24**, 372-6 (2000).
34. Shafi, R. et al. The O-GlcNAc transferase gene resides on the X chromosome and is essential for embryonic stem cell viability and mouse ontogeny. *Proc Natl Acad Sci U S A* **97**, 5735-9 (2000).
35. Zhao, W. et al. Jmjd3 inhibits reprogramming by upregulating expression of INK4a/Arf and targeting PHF20 for ubiquitination. *Cell* **152**, 1037-50 (2013).
36. Nishi, M. et al. A distinct role for Pin1 in the induction and maintenance of pluripotency. *J Biol Chem* **286**, 11593-603 (2011).
37. Nitzsche, A. et al. RAD21 cooperates with pluripotency transcription factors in the maintenance of embryonic stem cell identity. *PLoS One* **6**, e19470 (2011).
38. McDonel, P., Demmers, J., Tan, D.W., Watt, F. & Hendrich, B.D. Sin3a is essential for the genome integrity and viability of pluripotent cells. *Dev Biol* **363**, 62-73 (2012).
39. Kidder, B.L., Palmer, S. & Knott, J.G. SWI/SNF-Brg1 regulates self-renewal and occupies core pluripotency-related genes in embryonic stem cells. *Stem Cells* **27**, 317-28 (2009).

40. You, J.S. et al. SNF5 is an essential executor of epigenetic regulation during differentiation. *PLoS Genet* **9**, e1003459 (2013).
41. Pasini, D., Bracken, A.P., Hansen, J.B., Capillo, M. & Helin, K. The polycomb group protein Suz12 is required for embryonic stem cell differentiation. *Mol Cell Biol* **27**, 3769-79 (2007).
42. Pijnappel, W.W. et al. A central role for TFIID in the pluripotent transcription circuitry. *Nature* **495**, 516-9 (2013).
43. Liu, Z., Scannell, D.R., Eisen, M.B. & Tjian, R. Control of embryonic stem cell lineage commitment by core promoter factor, TAF3. *Cell* **146**, 720-31 (2011).
44. Ang, Y.S. et al. Wdr5 mediates self-renewal and reprogramming via the embryonic stem cell core transcriptional network. *Cell* **145**, 183-97 (2011).
45. Fidalgo, M. et al. Zfp281 functions as a transcriptional repressor for pluripotency of mouse embryonic stem cells. *Stem Cells* **29**, 1705-16 (2011).
46. Yu, H.B., Kunarso, G., Hong, F.H. & Stanton, L.W. Zfp206, Oct4, and Sox2 are integrated components of a transcriptional regulatory network in embryonic stem cells. *J Biol Chem* **284**, 31327-35 (2009).
